# Supplementary material for: Unraveling the processes shaping mammalian gut microbiomes over evolutionary time
Source: Nat Commun. 2017 Feb 23;8:14319. doi: 10.1038/ncomms14319 (PMC5331214; doi:10.1038/ncomms14319)
Supplement: Supplementary Information — Supplementary Figures, Supplementary Table, Supplementary Notes, Supplementary Discussion, Supplementary Methods and Supplementary References [file ncomms14319-s1.pdf]

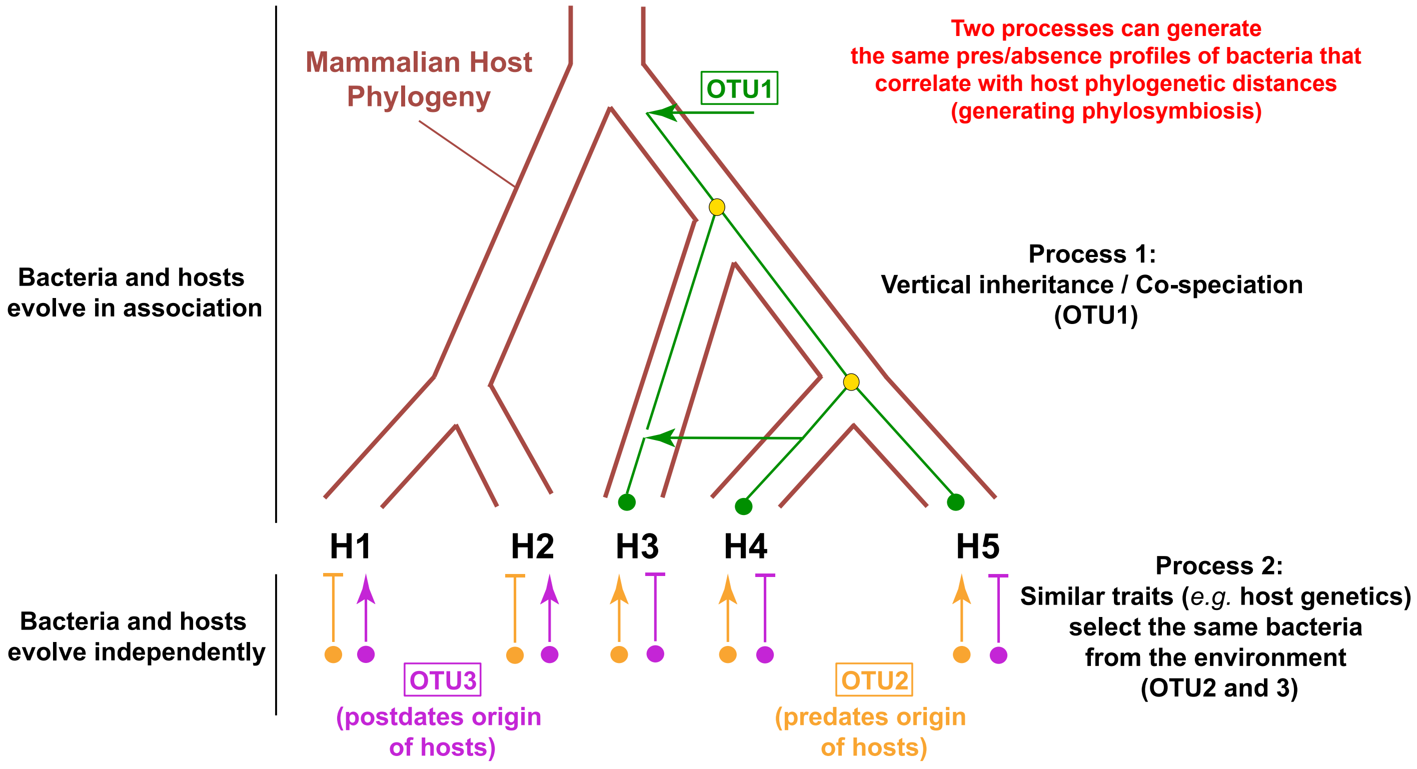

Supplementary Figure 1 | Hypothetical illustration of the processes that can generate phylosymbiosis. The host phylogenetic tree is represented in brown as bifurcating tubes. Three examples of host-bacteria associations are shown. All three are correlated with host phylogenetic distances and can contribute in generating a signal of phylosymbiosis. The evolutionary trajectory of OTU1, which evolves in close association with hosts, is represented in green lines. OTU1 undergoes co-speciation with the host, but may experience host swap along host evolution. The degree of topological congruency between the symbiont and host phylogenies sheds light on the dynamics of bacteria-host associations. If topological congruency is high, the rate of co-speciation is high, meaning that bacteria have restricted host-specific ranges and co-diversify with hosts. If topological congruency is low, the rate of host-swap is high, meaning that bacteria have wide host ranges and can be easily transferred. Weak topological congruency is also expected for OTU2 and OTU3, which are evolving independently from hosts: the origin of OTU2 predates the origin of hosts in which it is observed, and the origin of OTU3 postdates the origin of hosts. However, their distribution across hosts is correlated with host phylogenetic distances: hosts 3, 4 and 5 select OTU2 but not OTU3, while hosts 1 and 2 select OTU3 but not OTU2. Consequently, while these three presence/absence patterns significantly correlate with host phylogeny (*e.g.* with a Permanova test, as done in Figure 1B in the Main Text), the processes generating these patterns are very different. To distinguish between these scenarios, a modelling of symbiont evolution along the host tree accounting for both the information in the symbiont phylogeny and the rates of co-speciation and host-swap is necessary to compute the most likely scenario.

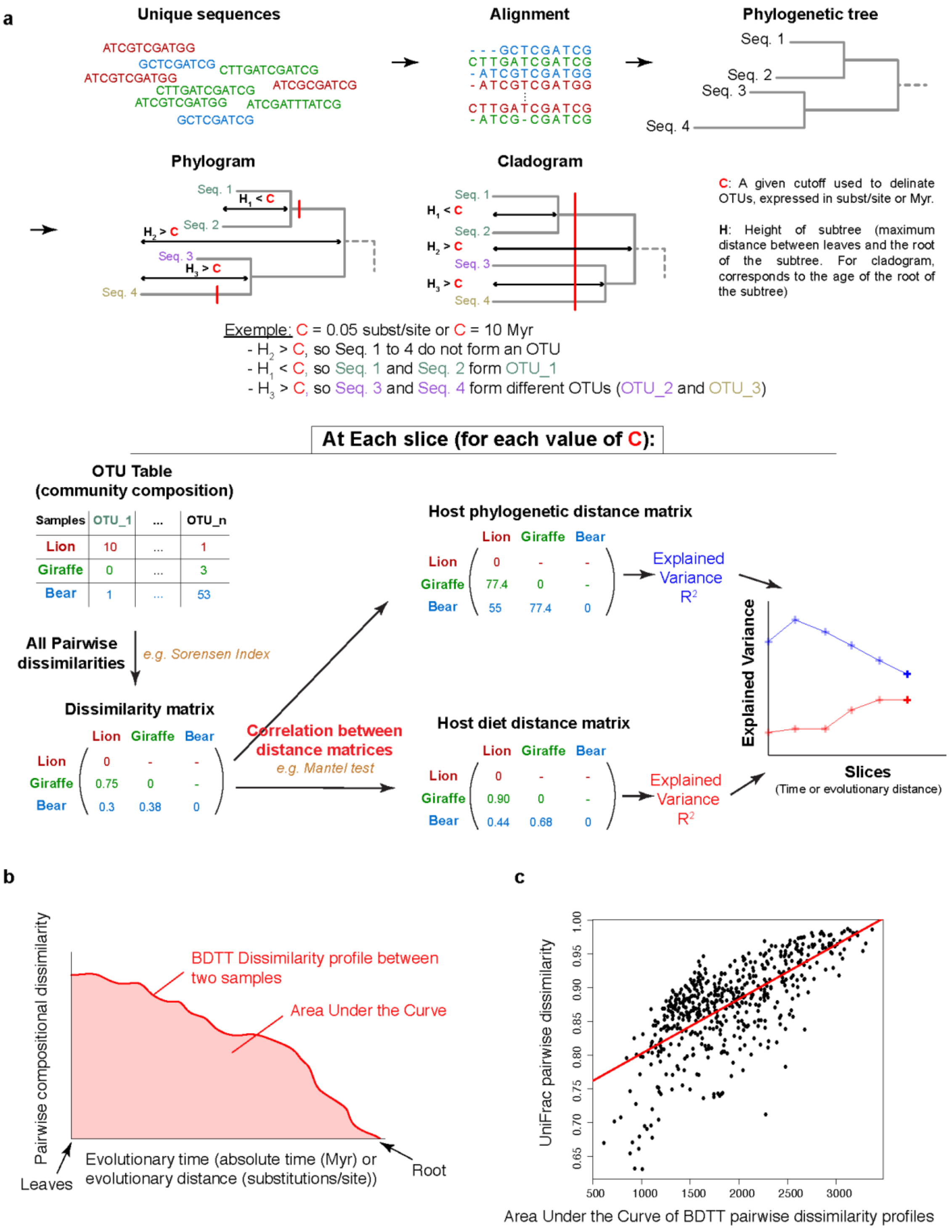

Supplementary Figure 2 | Principles and validation of the BDTT approach.
a: Description of the BDTT procedure (see Main text (Results & Methods sections) for a complete description). **b, c:** BDTT decomposes the UniFrac. We report a comparison between UniFrac dissimilarities and integrated BDTT profiles of dissimilarity. **b:** hypothetical BDTT dissimilarity profile for two communities, for which the Area Under the Curve (AUC) is computed. **c:** AUC for all pairwise BDTT profiles were computed on our mammalian dataset. AUC (X-axis) is plotted against the UniFrac dissimilarity (Y-axis).

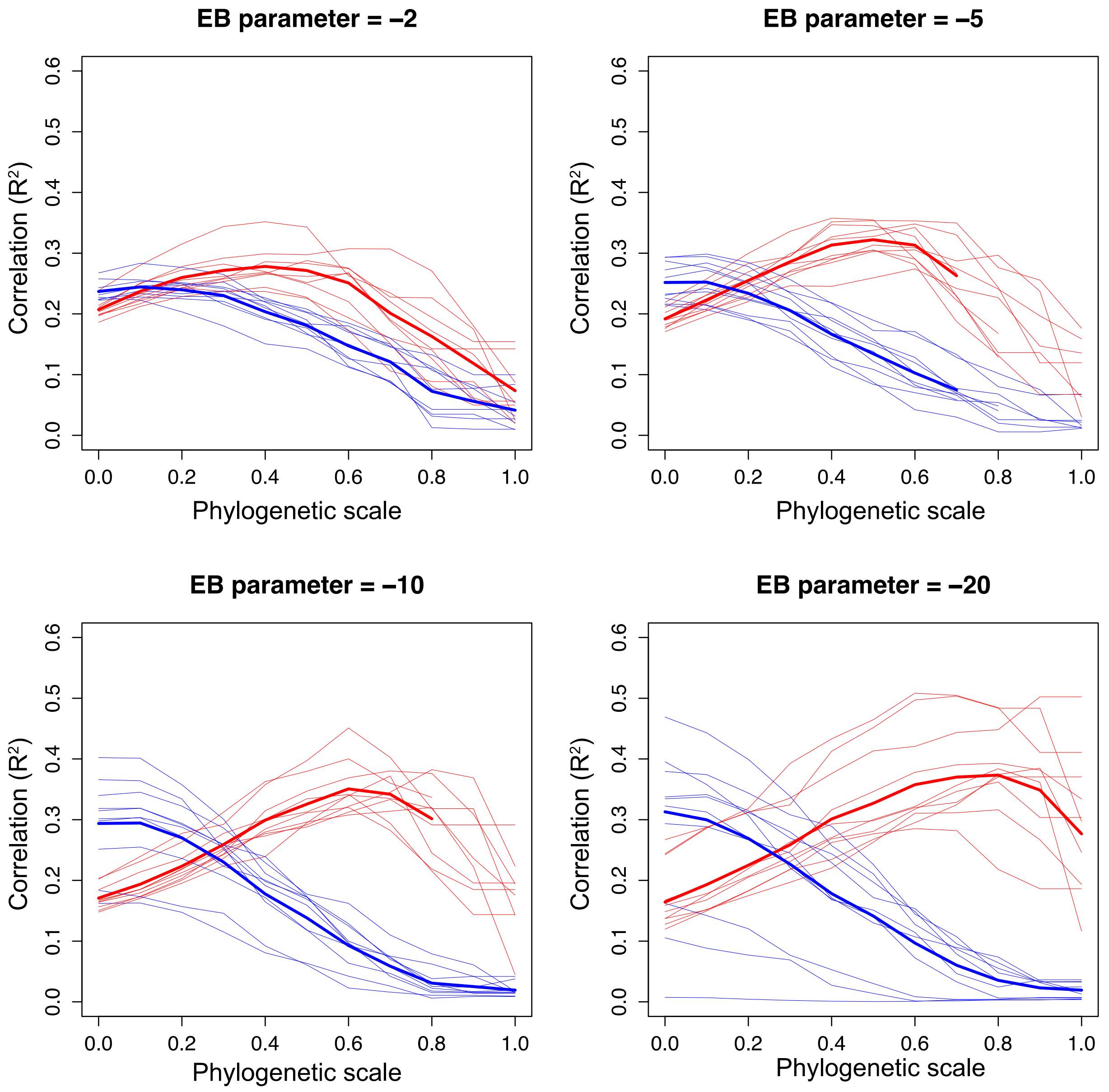

Supplementary Figure 3 | BDTT captures phylogenetic scale disparities between the effects of environmental factors on community compositions. The four panels depict the correlation between beta-diversity and environmental distances (defined by distances between fixed environmental values of communities) (Y-axis) along the phylogenetic time scale (X-axis, left: tips of the tree; right: root of the tree). Each panel corresponds to different pairs of simulated environmental preferences (traits) of species: the blue correlation profile corresponds to environmental preferences of species simulated under a BM model, while the red correlation profile corresponds to environmental preferences of species simulated under an EB model. The EB parameter on top of each panel indicates the rate at which trait evolution slows through time along the phylogeny. The more negative this parameter is, the faster the rate of trait evolution slows from the root, creating trait disparities mostly in the deepest regions of the tree. Light colours represent the correlation profiles of each simulated tree averaged across the 10 simulated sets of traits. Plain colours represent profiles averaged over the 10 phylogenies (*i.e.* mean correlation profile over all simulations).

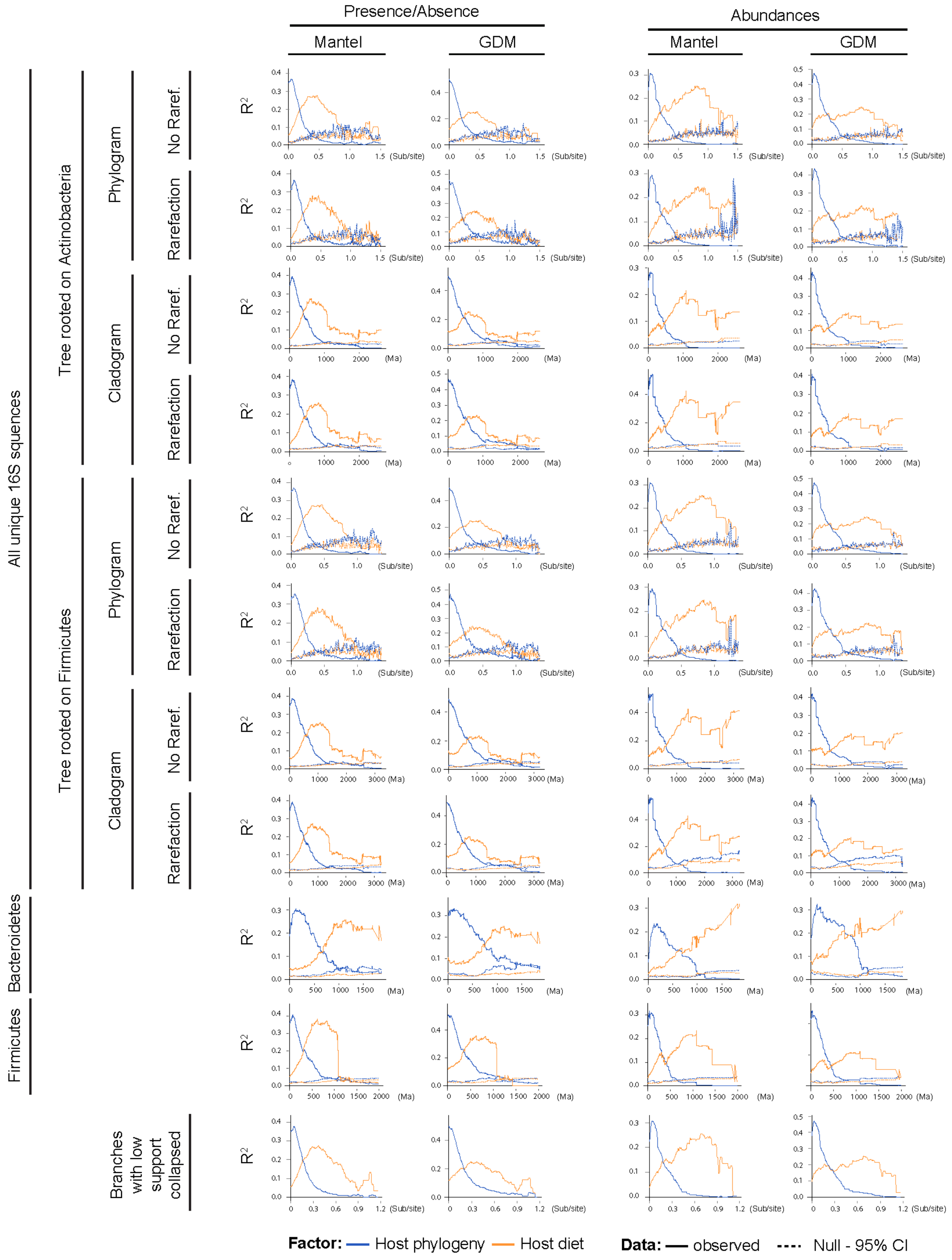

Presence/Absence
Abundances
Mantel
GDM
Mantel
GDM
All unique 16S squences
Tree rooted on Actinobacteria
Tree rooted on Firmicutes
Phylogram
Cladogram
No Raref.
Rarefaction
Bacteroidetes
Firmicutes
Branches with low support collapsed
R^2
(Sub/site)
(Ma)
Factor: Host phylogeny Host diet
Data: observed Null - 95% CI

Supplementary Figure 4 | Control experiments for the scale disparity between the effects of host phylogeny and diet on mammalian gut microbiome compositions. We performed correlations between microbiome compositions and host phylogeny and diet using BDTT in different conditions: (1) with all 16S sequences or 16S restricted to Firmicutes or Bacteroidetes sequences; (2) with a rooting of the bacterial phylogenetic tree on Actinobacteria or on Firmicutes; (3) with branch lengths expressed in "time" units or in substitutions/site and (4) using rarefied or non-rarefied OTU tables. We also controlled for the influence of topological uncertainties in the bacterial tree on the construction of OTU tables along the tree. Conclusions drawn in Figure 1A hold true in these different control conditions.

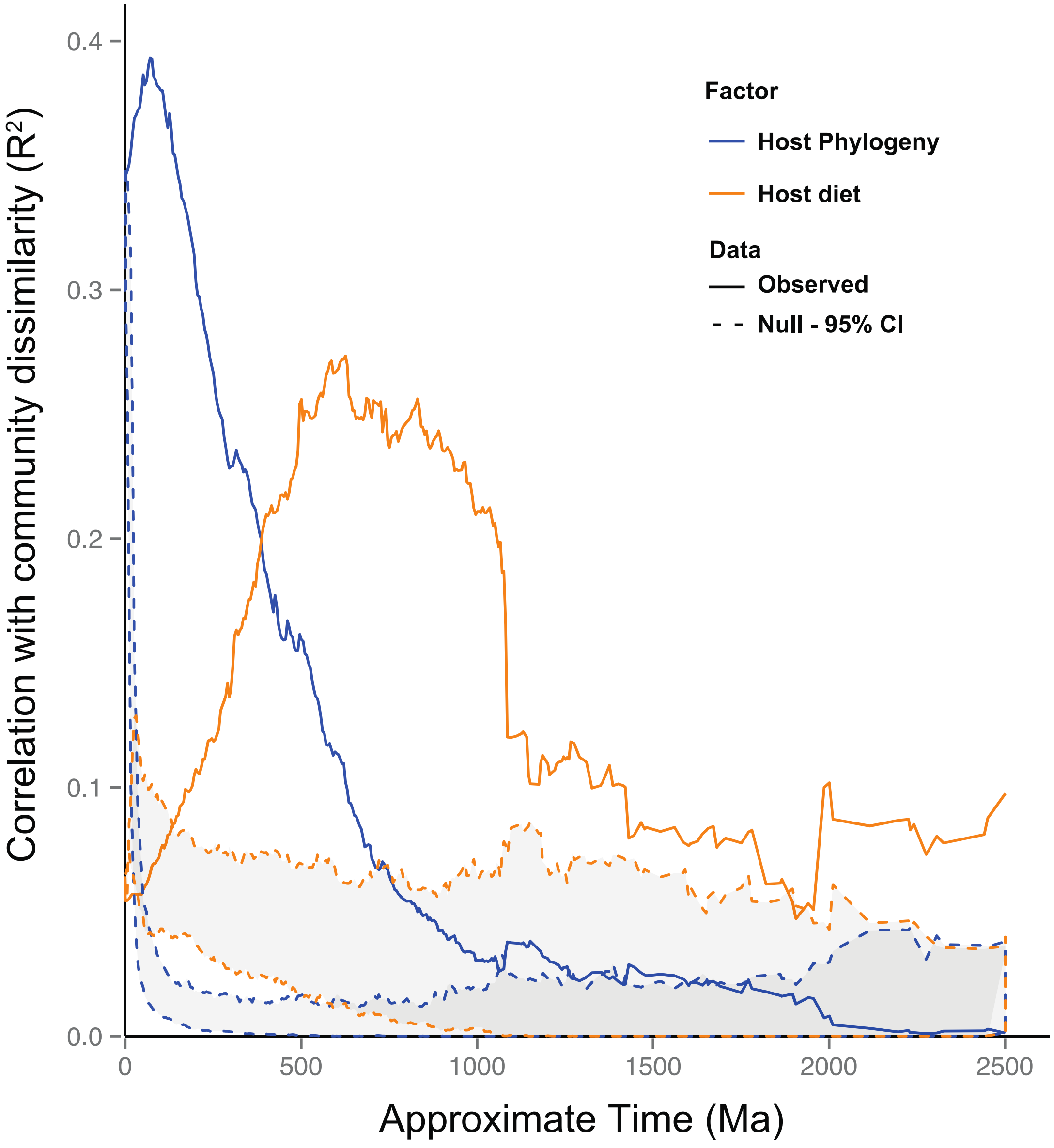

Supplementary Figure 5 | Correlation profiles between microbiome composition and host phylogeny and diet expected under a null model. The null model controls for the effect of the hierarchical structure of the phylogenetic tree on correlations with host phylogenetic (dark blue) and dietary (orange) distances. The distributions of null correlation profiles are represented in the form of a 95% null envelope. The time scale of the bacterial phylogeny is represented in the X-axis. Observed correlation profiles are identical to those in Figure 1A (Main Text). See section 2.3.4 in Supplementary Discussion for more details about the null model.

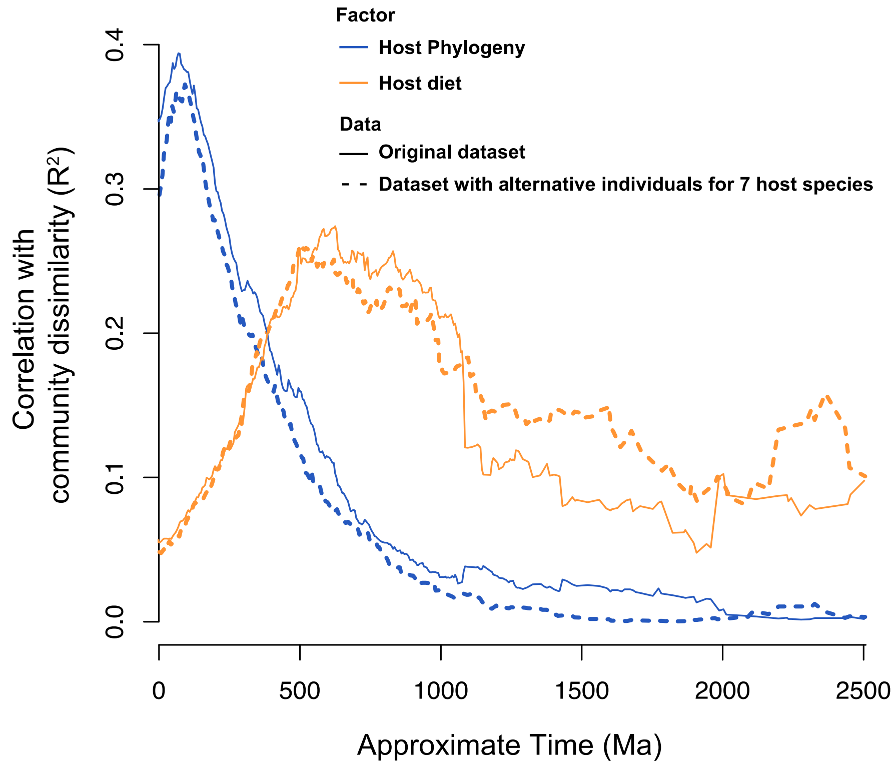

Supplementary Figure 6 | Control for the impact of intra-host variability on the scale disparity between the effects of host phylogeny and diet. The BDTT analyses were run as in Fig. 1A. The plain blue and orange lines show the original correlation profiles with host phylogeny and diet, respectively (Fig. 1A). The dashed lines show the correlation profiles with both factors that we obtained when using the gut microbiome of alternative individuals for 7 host species. This control confirms that the intra-host compositional variability is much weaker than the inter-host compositional differences and that our main conclusions regarding associations between microbiomes and host phylogeny and diet drawn in our manuscript are not biased by our choice of individuals within each host species.

Supplementary Figure 7 | The high correlation with host phylogeny in recent regions of the bacterial tree does not depend on the fine granularity of the matrix of host phylogenetic distances. The top left panel shows the distribution of all pairwise host distances in time units (Ma) between our 33 mammals. The other panels are replicates of Fig. 1A, using different granularities for the matrix of host phylogenetic distances, from fine-grained (top right panel) to coarse-grained (bottom panels) matrices. PHPD: Pairwise Host Phylogenetic Distance. For a given plot, all PHPDs below a given distance threshold are set to 0, decreasing the granularity of the original distance matrix. When the granularity of the host phylogenetic distance matrix is getting coarse, the correlation with gut microbiome compositions is decreasing, as expected. However, the maximum of this correlation is not shifting towards more ancient regions of the bacterial tree, and the scale disparity between the effects of host phylogeny and diet is still observed.

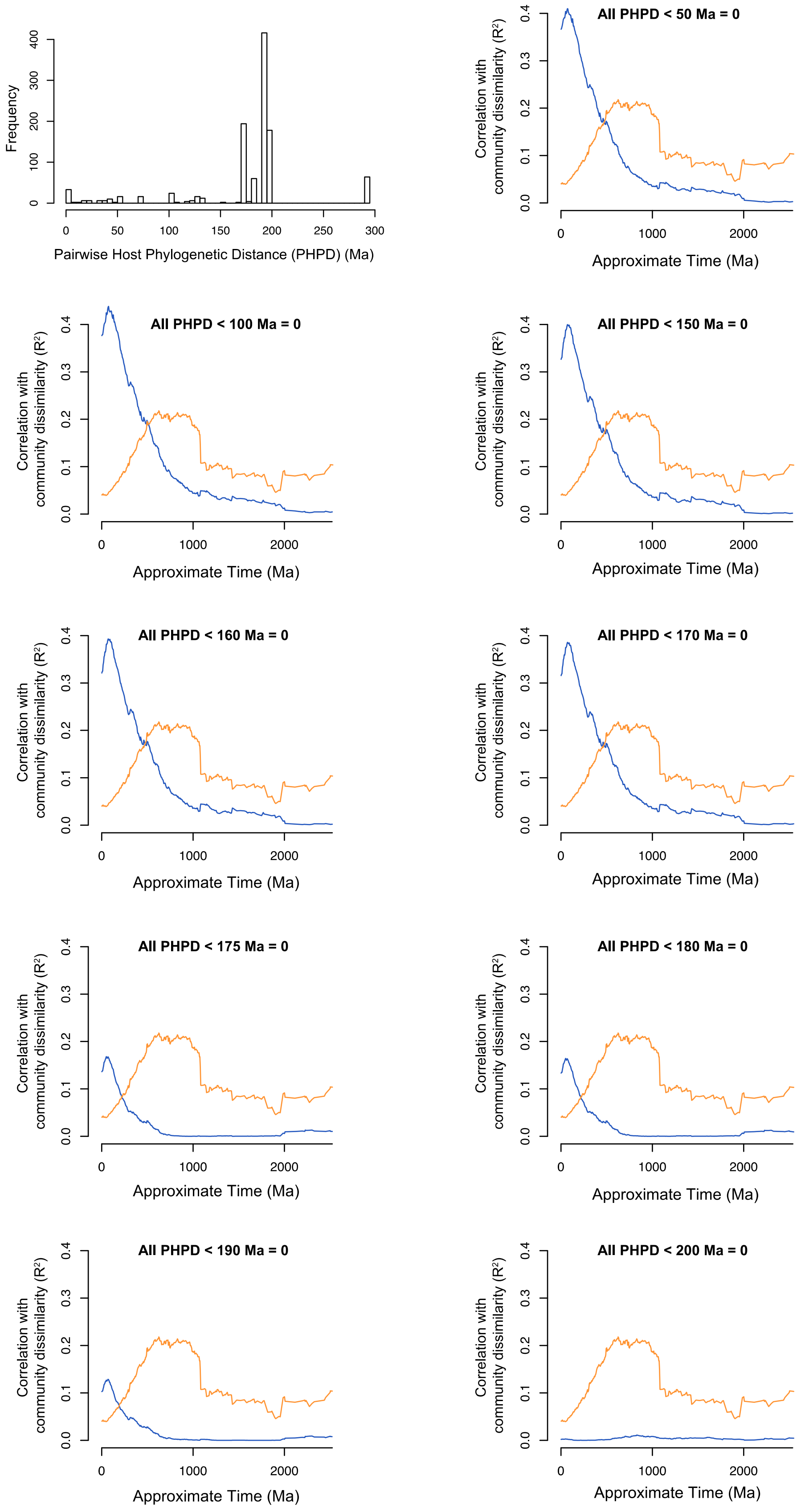

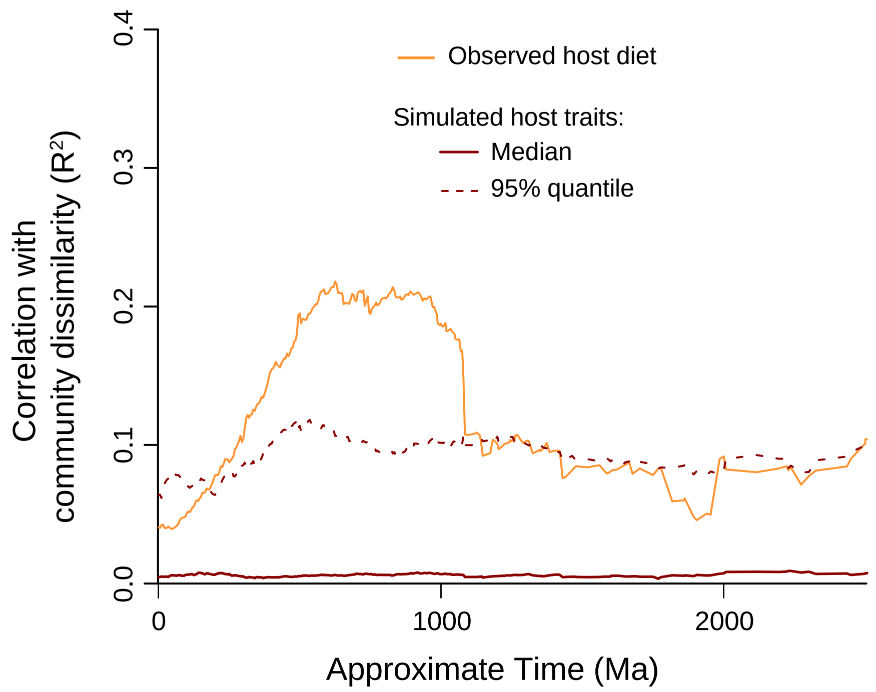

Supplementary Figure 8 | The peak in correlation between diet and gut microbiome compositions at ancient timescales is not simply an echo of phylogenetic history written in diet. We simulated phylogenetically-conserved traits that evolved along the mammalian phylogeny at the same rate as diet does (100 replicates), and compared the correlation profiles between these simulated traits and microbiome compositions with the correlation profile obtained with observed diets. The distributions of simulated correlation profiles are represented in the form of a 95% null envelope. The dark red plain line connects the medians of these distributions. The dark red dashed line connects the 95% quantiles. The original correlation profile with observed diets is in orange and is the same as in Fig. 1A. The high correlation with observed diets at ancient timescales is significantly higher than the null, showing that there is a genuine signal associated with diet that is independent from the host phylogenetic history written in diet.

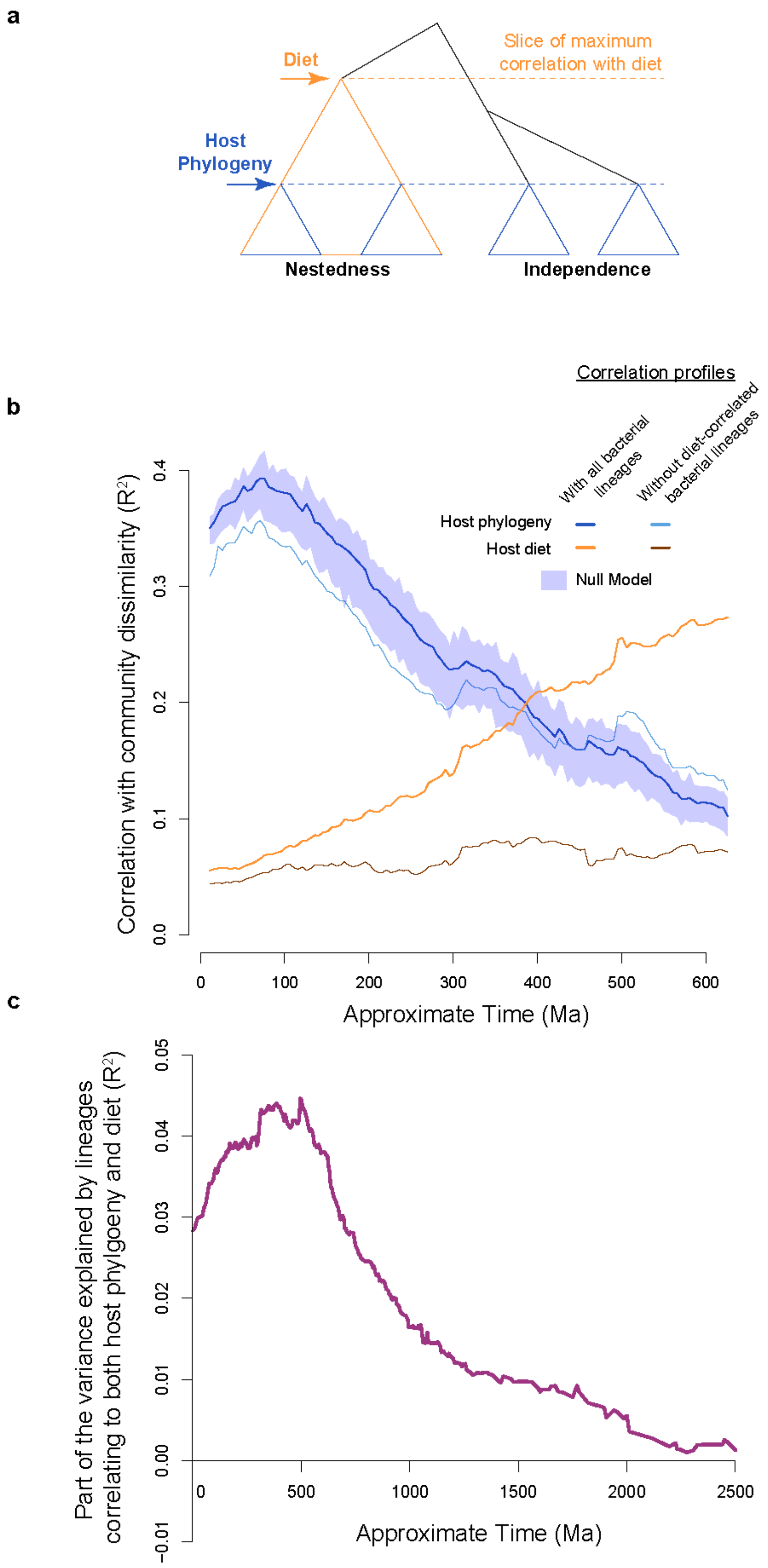

Supplementary Figure 9 | Bacterial lineages correlating with host phylogeny and diet lowly overlap. a, Diagram depicting the possible relationships between bacterial lineages that are significantly correlated with host phylogeny and/or diet. Lineages that are correlated to host phylogeny at shallow phylogenetic scales may or may not be nested within lineages correlated to diet at higher scales. **b**, Correlations with host phylogeny are weakly affected when lineages correlated to diet at the slice where the community-level correlation with diet is maximal (Fig. 1A) are removed. **c**, Variance in microbiome composition explained by the covariance between dietary and phylogenetic distances. The overall contribution of the co-variance of host phylogeny and diet in shaping microbiome compositions is weak (the maximum observed R^2 value is about 4%).

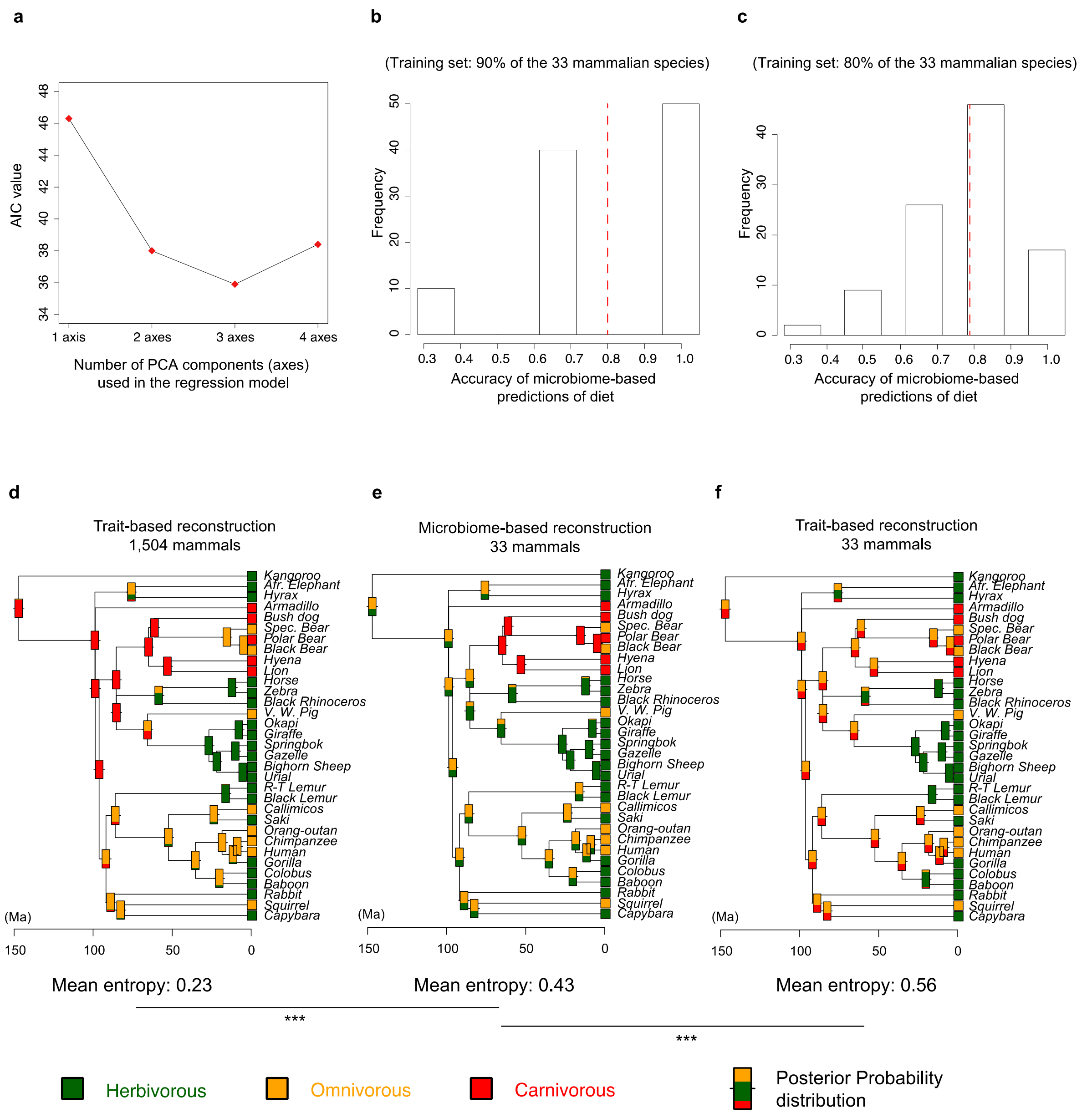

Supplementary Figure 10 | Microbiome-based method to infer ancestral host diets. **a**. Model selection (using AIC) for the multinomial logistic regression used to predict diet from microbiome compositions. Here, AIC selects the regression model containing only three independent predictors (components of the PCA computed from gut microbiome compositions, see Figure 2). **b, c,** Cross-validation experiments performed to measure the accuracy of microbiome-based predictions of diet. 100 cross-validations were performed, by defining the *Training set* of mammalian gut microbiome with either 90% or 80% of the total number of hosts. The red dotted line represents the mean accuracy over the 100 simulations. **d, e, f,** Trait-based and microbiome-based inferences of ancestral diets in mammals. Microbiome-based predictions are significantly more precise than trait-based predictions performed with a similar taxonomic sampling. Two-tailed Wilcoxon rank-sum tests. ***: *p-value* < 0.001.

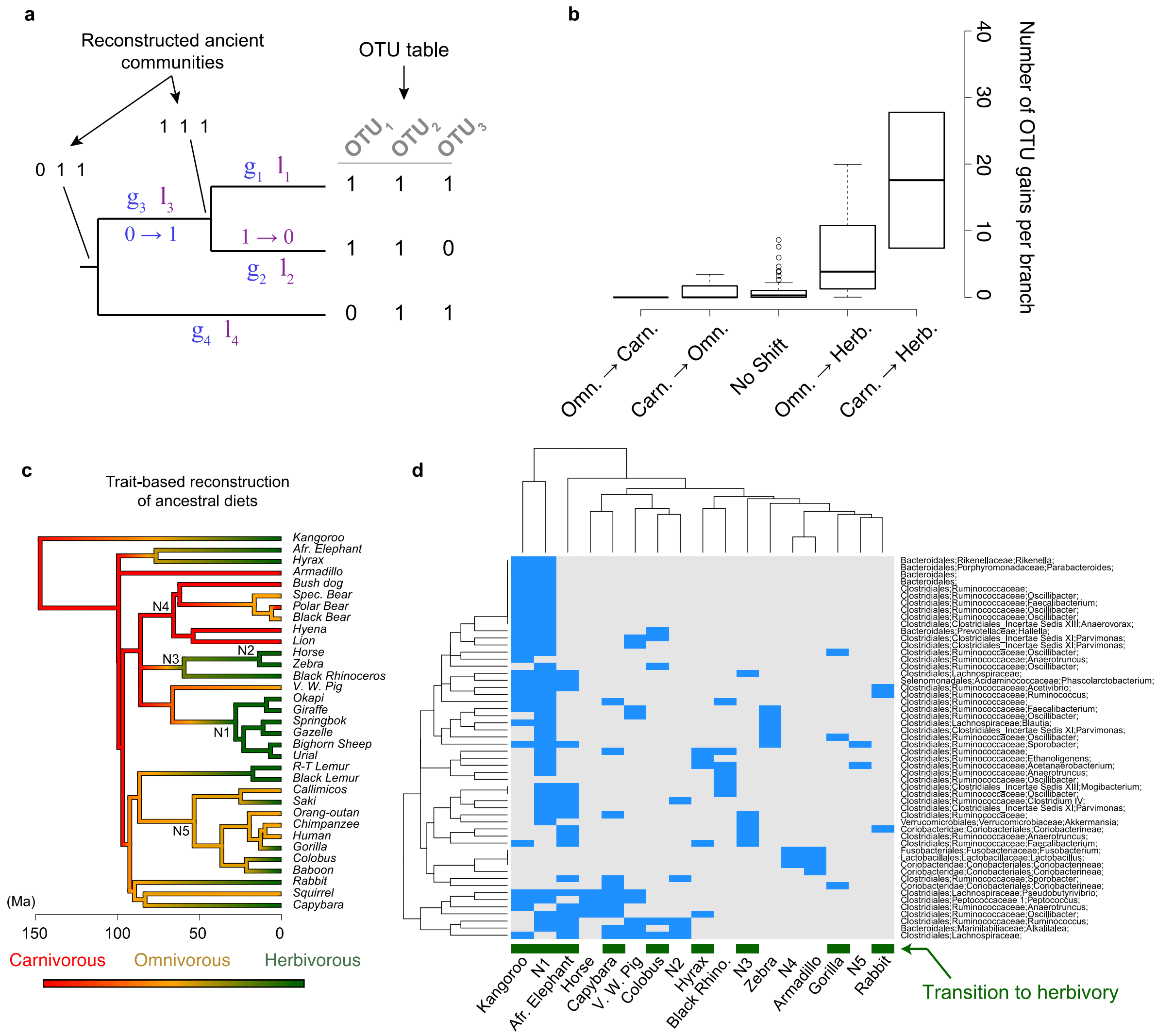

Supplementary Figure 11 | Multiple convergent acquisitions of herbivorous-specific bacterial lineages during dietary transitions towards herbivory. **a**, Detailed diagram of the reconstruction of ancestral microbiome. *g*: rate of OTU gain. *l*: rate of OTU loss. Both gain and loss rates are branch-specific. 0 -> 1 means that the OTU is inferred to be gained along the branch. ML rate estimates allow us to compute posterior probabilities of ancestral OTU presence for all OTUs at each node. **b,** Boxplot of the number of gains per type of dietary transition. **c**, Reference trait-based inference of ancestral diets using 1,534 mammals (same as in Figure 2C). This plot is only for illustration purposes, to visualize the locations of transitions towards herbivory. **d**, Heatmap of the OTU gains across all mammalian lineages. Branches N1 to N5 are shown in **c**. This heatmap highlights the convergent gain events of several OTUs during adaptation to herbivory.

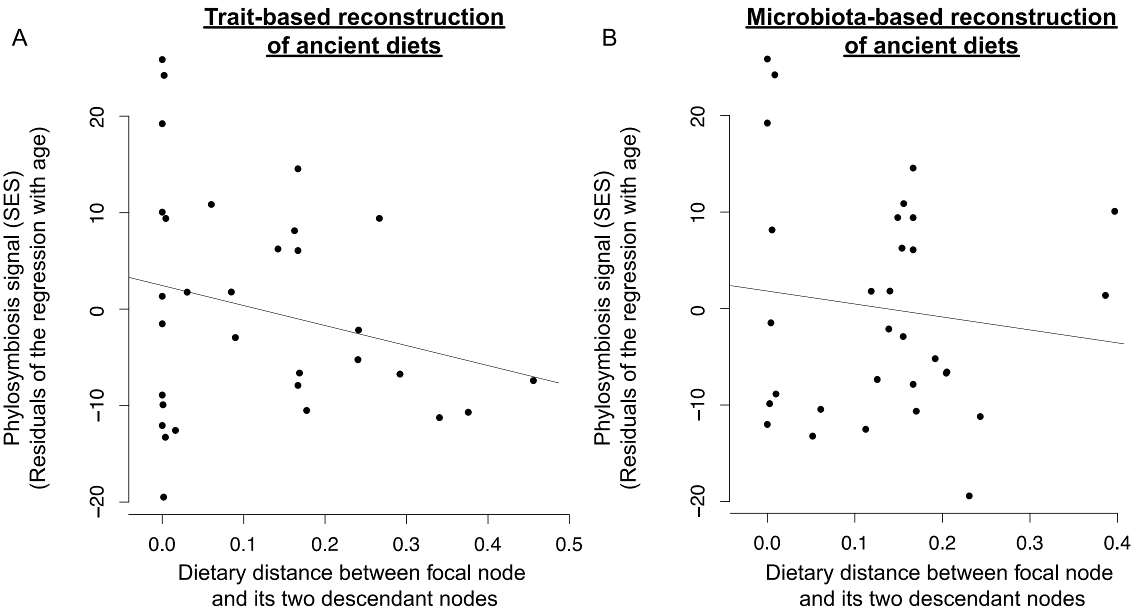

Supplementary Figure 12 | The effect of dietary shifts on the phylosymbiosis signal. These plots represent the relationship between the magnitude of phylosymbiosis (Y-axis) and the extent of dietary shift at each node. Phylosymbiosis at each node is expressed as the residual of the regression between phylosymbiosis and age at this node (see also Results – The phylosymbiosis is strong in mammals and Figure 3). Dierary shifts were computed using ancestral diets reconstructed both with the trait-based approach using the dietary data of 1,534 mammals and with the microbiome-based approach (see Methods – Reconstruction of ancestral diets). Slope values were negative but weak (r = -0.15 and r = -0.1 when ancestral diets were predicted with the trait-based and microbiome-based methods, respectively).

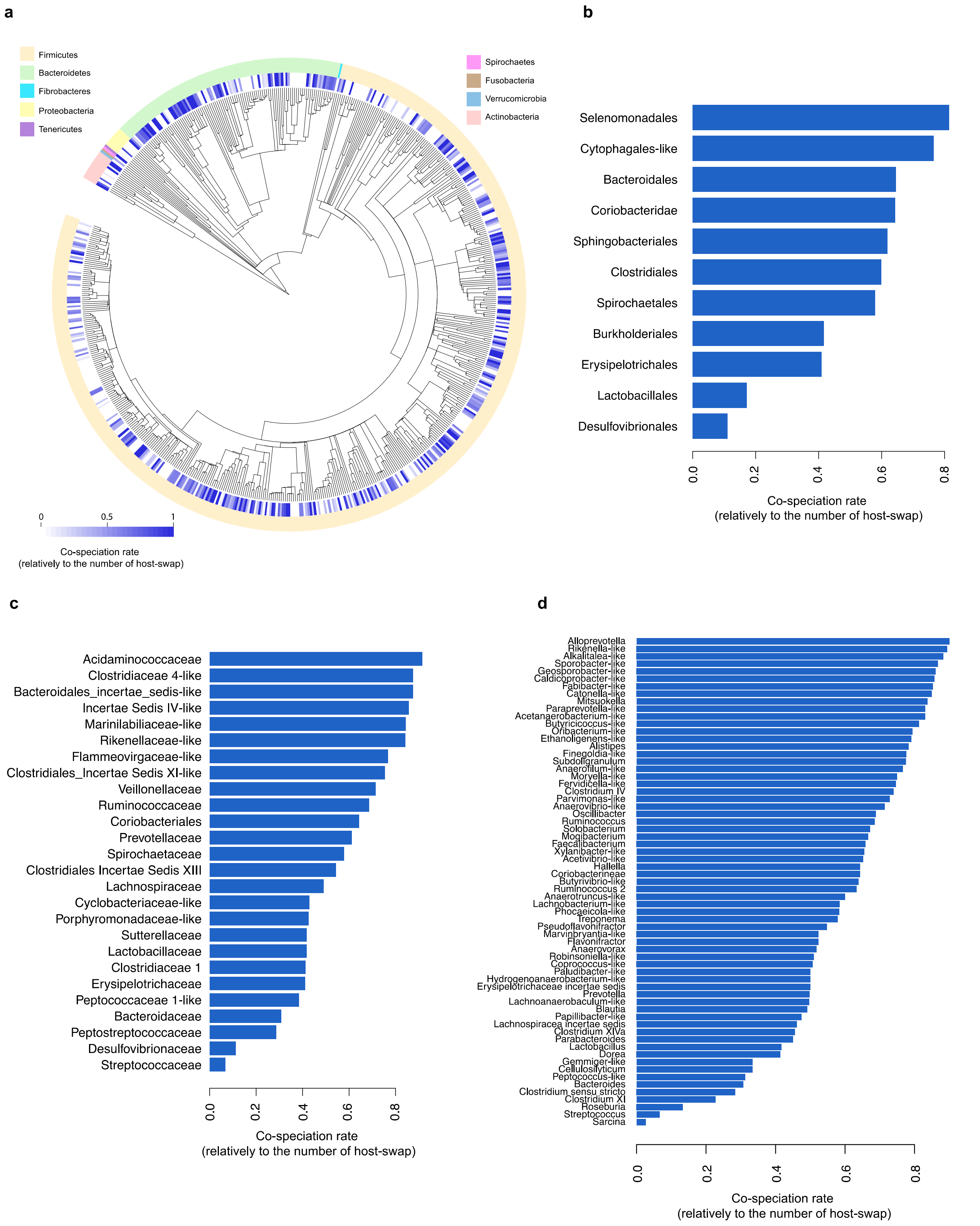

Supplementary Figure 13 | The phylogenetic signal of co-speciation between bacterial lineages and their mammalian host. At the phylogenetic scale that we used to perform measurements of co-speciation rates (see Methods), 620 bacterial lineages are present in at least 4 hosts. **a,** The phylogeny of the 620 representative sequences of these OTUs is represented. The

outer circle depicts the co-speciation rates of all OTUs, along a colored gradient (white: only host-swap events, blue: only co-speciation events). **b, c, d**, Co-speciation rates by bacterial order (b), family (c) and genus (d). For an OTU, the co-speciation rate is defined as the number of co-speciation events divided by the total number of co-speciation and host-swaps. Within a given bacterial taxonomic rank, co-speciation rates were computed for all OTUs. Average co-speciation rates are reported on the X-axis. Only bacterial ranks having a minimum of 2 OTUs are reported here.

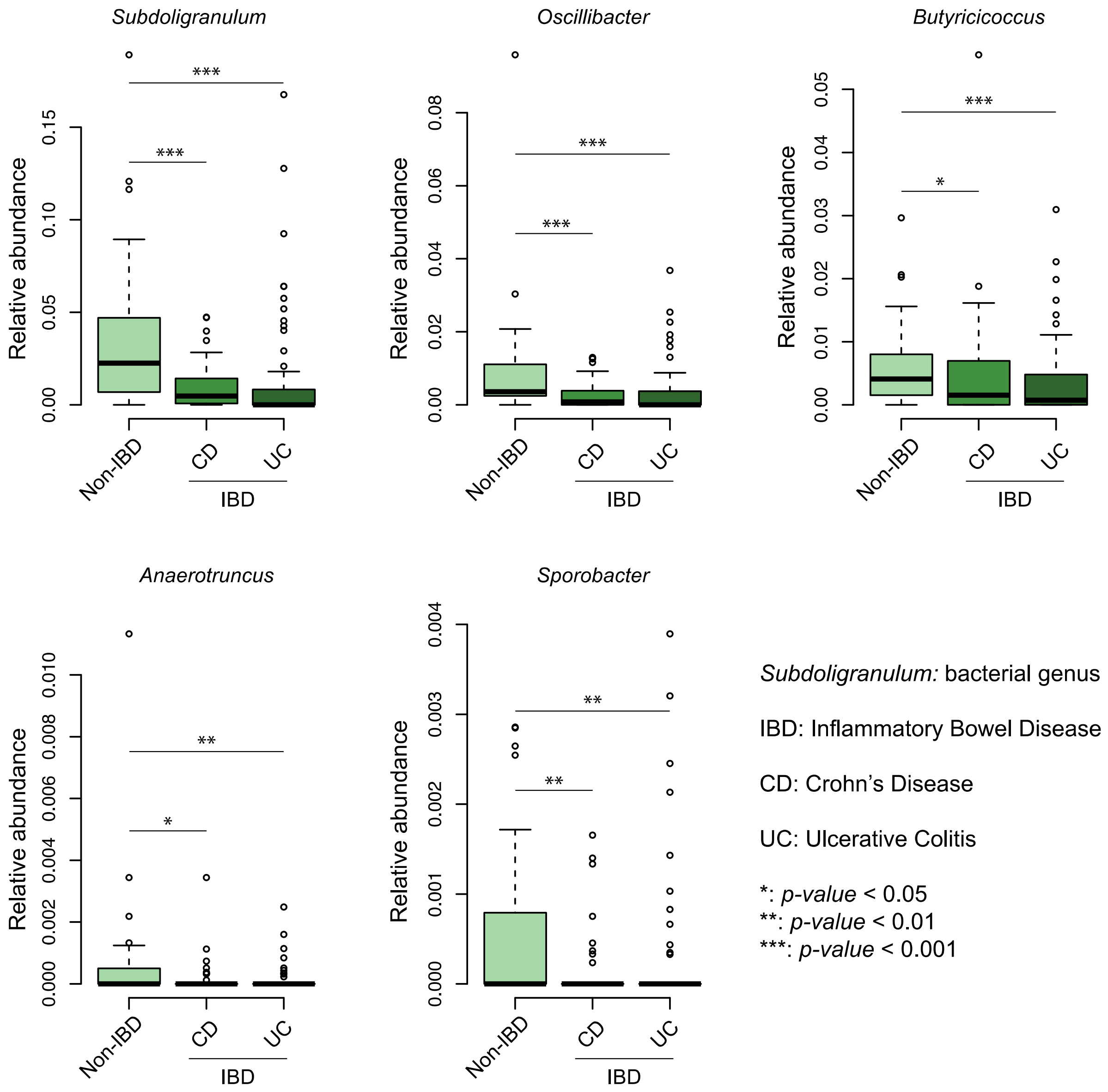

Supplementary Figure 14 | Co-speciating bacterial genera present in humans and that are strongly correlated with inflammatory bowel diseases (IBD). 20 highly co-speciating OTUs (co-speciation rate ≥ 0.8) are present in humans. 13 belong to five bacterial genera that were previously found to be accurate predictors of IBD (see Supplementary Discussion section 2.9). The data shown here were extracted from this previous study. Two-tailed Wilcoxon rank-sum tests were used to compare the means of relative abundance between populations.

Supplementary Table

Species	Plant	Seed	Nectar	Fruit	Carcass	Vertebrate - unknown	Vertebrate - fish	Vertebrate	Invertebrate
Afr. Elephant	100	0	0	0	0	0	0	0	0
Armadillo	0	0	0	20	0	0	0	0	80
Baboon	80	0	0	0	0	10	0	0	10
Bighorn Sheep	100	0	0	0	0	0	0	0	0
Black Bear	30	0	0	50	0	0	10	10	0
Black Lemur	70	0	0	30	0	0	0	0	0
Black Rhinoceros	100	0	0	0	0	0	0	0	0
Bush dog	0	0	0	0	0	0	0	100	0
Callimicos	0	0	0	50	0	10	0	0	40
Capybara	90	0	0	10	0	0	0	0	0
Chimpanzee	30	0	0	60	0	0	0	0	10
Colobus	70	0	0	30	0	0	0	0	0
Human	30	0	0	60	0	0	0	0	10
Gazelle	100	0	0	0	0	0	0	0	0
Giraffe	100	0	0	0	0	0	0	0	0
Gorilla	90	0	0	10	0	0	0	0	0
Horse	100	0	0	0	0	0	0	0	0
Hyena	0	0	0	0	0	0	0	100	0
Hyrax	100	0	0	0	0	0	0	0	0
Kangoroo	100	0	0	0	0	0	0	0	0
Lion	0	0	0	0	10	0	0	90	0
Okapi	100	0	0	0	0	0	0	0	0
Orang-outan	0	0	0	80	0	10	0	0	10
Polar bear	0	0	0	0	0	0	10	90	0
Rabbit	100	0	0	0	0	0	0	0	0
Ring-Tail Lemur	20	0	0	80	0	0	0	0	0
Saki	30	0	10	30	0	0	0	30	0
Spectacled bear	20	0	0	80	0	0	0	0	0
Springbok	100	0	0	0	0	0	0	0	0
Squirrel	30	20	0	20	0	10	0	0	20
Urial	100	0	0	0	0	0	0	0	0
Visayan Warty Pig	60	10	0	0	10	10	0	0	10
Zebra	100	0	0	0	0	0	0	0	0

Supplementary Table 1: Dietary information extracted from the EltonTraits database for the 33 mammals investigated in this study.

Supplementary Methods

Phylogenetic reconstructions

We used PATHd8 [1] to produce a cladogram from the ML tree reconstructed by FastTree. We set the origin of Cyanobacteria at >2.5 Ga Ma [2]. The divergence between Rickettsiales and the rest of Alphaproteobacterial sequences was set at >1.6 Ga, since the ancestor of the mitochondrion was likely a sister to Rickettsia, and the eukaryotic ancestor is likely to be older than 1.6 Ga [3]. We calibrated the divergence between Chlorobium and Bacteroidetes at >1.64 Ga, since Chlorobium is the sister clade of Bacteroidetes and Chlorobium-specific biomarkers were found in rocks at 1.64 Ga [4]. Finally, the divergence between Chromatiales and other Gammaproteobacterial sequences at set at >1.64 Ga, since purple sulphur bacterial biomarkers were also found in rocks at 1.64 Ga [4]. A maximum divergence time of 3.8 Ga was set at the root of the tree. As there are few time calibrations in comparison with the number of leaves in the tree, the calibrations have a very little influence on the computation of divergence times, which are very similar to those obtained when assuming a strict molecular clock.

Validation of BDTT on simulated data

Below, we describe in more details the simulation framework that we used to validate our approach on simulated data and show that BDTT can extract signals of phylogenetic scale disparity between the effects of different factors on community compositions.

i) Phylogenies – We simulated 10 pure birth phylogenies of 200 species using the function *bdtree* implemented in the geiger R package.

ii) Traits – For each phylogeny, we simulated 10 independent sets of continuous traits using classic models of trait evolution. Because we aimed to test if BDTT was able to disentangle factors acting at different phylogenetic scales, we simulated traits that harbour phylogenetic signals at different scales. To do so, we used an early burst (EB) model of evolution [5,6]. This model is an extension of the classic Brownian Motion (BM) model of trait evolution and allows the rate of evolution to exponentially decrease with time as radiation of species proceeds [6]. This exponential decrease is parameterized and can be slow or fast. When the rate at which trait evolution slows through time (called r) equals 0, the EB model is reduced to a BM model. When

r is negative, the rate of trait evolution progressively slows toward the tips of the phylogeny (= toward the present), so that most of the phylogenetic signal of the trait is concentrated in early regions of the phylogeny. As a consequence, the use of multiple traits with different values for the *r* parameter allows us to simulate traits that harbours phylogenetic signal at different phylogenetic scales. We simulated traits according to a classic BM and four types of EB models with 4 values of *r* (-20, -10, -5, -2). Overall, we simulated 100 replicates per each combination of BM and EB models: 10 different phylogenies × 10 pairs of simulated traits for all species.

iii) Community Assembly – The rationale of our simulation experiment is to test BDTT on communities assembled under known processes. Here, we assembled species communities under environmental filtering, *i.e.* selection of species according to the match between their environmental preference (= trait values) and the fixed environmental conditions of communities. A given species will remain in the community if its trait values are close enough to the optimal trait values required for being part of this community. To do so, we take advantage of a recently published community assembly simulation platform named 'VirtualCom' implemented in R [7]. VirtualCom is an individual-centred model that is initially designed to assemble communities based on a phylogeny and three sorts of filter: (i) environmental (the probability of an individual entering the community through the environmental filter is higher if its environmental preference is close to the community environmental value), (ii) competitive (the probability of an individual entering the community through the competitive filter is higher if there is no competitor present in the community) and (iii) reproductive (the probability of an individual entering the community through the reproduction is higher if conspecifics are already present in the community). We modified VirtualCom to (i) incorporate our own simulated species traits (see above) and (ii) to account for a second environmental filter. To do the last modification, we assumed that the two traits are independent, and so that the effect of the combined environmental gradient on the probability of on individual to enter in a community was simply the product of the two independent environmental filters. To initialize model parameters, we tested a broad range of values and selected those that yielded stable communities over time (L. Gallien, personal communication). In our case, we fixed these parameters:

- Niche breadth = 0.5
- Strength of the environmnetal filetr (B_{env} [7]) = 0.5

- Strength of the reproduction filter (B_{abun} [7]) = 0.5

- Strength of the competition filter (B_{comp} [7]) = 0, because the competition process is not needed to test BDTT.

- Number of simulated years = 30

- Carrying capacity = 300

In each case, we simulated 25 environmental conditions for 25 communities, with these conditions being regularly spaced within a fixed environmental space (the range of the environmental space was set equal to the range of centred and reduced simulated trait values). We assembled communities with a combination of two traits: a classic BM and one of the four different EB model (so four different pairs of simulated traits).

iv) BDTT – For each replicate, we carried out a BDTT analysis, producing profiles of beta-diversities along the phylogenetic time scale. We used the Sorensen metric to measure beta-diversities at each time slice, and we correlated all these beta-diversity matrices to matrices of environmental distances using Mantel tests (as in the main text of the article, see Methods – β-diversity Through Time and Phylogenetic clustering; Methods – Measuring correlation profiles with diet and host phylogeny)

Supplementary Notes

Supplementary Note 1. BDTT phylogenetically decomposes UniFrac

Phylogenetic β-diversity metrics such as UniFrac [8] are more precise for describing community composition than taxonomic β-diversity metrics because they account for shared evolutionary history between lineages/OTUs to measure community dissimilarity. Here, we used the mammalian dataset that we analysed in this study to perform comparisons between UniFrac dissimilarities and integrated BDTT profiles (we call a 'BDTT profile' a vector of pairwise compositional dissimilarities between two communities computed along the bacterial phylogenetic tree (if the tree is time-calibrated, the dissimilarities are computed over all time slices). Both methods make use of the phylogenetic tree of all sequences across all communities, but BDTT uses taxonomic β-diversity metrics to compute multiple compositional dissimilarities that are time-dependent [9] (see Supplementary Fig. 2). For all pairs of communities, we recorded the profile of β-diversities along all phylogenetic slices defined along the tree, and computed the integral of this profile, representing the integrated BDTT profile over the tree (Supplementary Fig. 2b). We show that the integrated BDTT profile correlates highly and positively to UniFrac (with the root of the tree on Actinobacteria and using the rarefied dataset: Pearson's r = 0.78, *p-value < 0.001*; with the root on Firmicutes: Pearson's r = 0.89, *p-value < 0.001*; see Results – Phylogenetic decomposition of community dissimilarities and Supplementary Fig. 2). We conclude that, as UniFrac, BDTT captures the phylogenetic component of the β-diversity between samples. However, BDTT possesses the advantage of providing a phylogenetic decomposition of this β-diversity metric that can be used to detect at which phylogenetic scales a given factor influence the most community compositions. Here, we applied BDTT to our mammalian gut microbiota dataset. However, we stress again that BDTT can be applied to any type of community, both at the microorganism and macroorganism scales.

Supplementary Note 2. BDTT captures scale disparities on simulated data

We run BDTT on simulated datasets to evaluate its ability to disentangle the effect of different factors that shape community assembly at different phylogenetic scales (see Results – Phylogenetic decomposition of community dissimilarities and Supplementary Methods – Validation of BDTT on simulated data). Correlation profiles between community dissimilarities

and environmental distances show that BDTT is able to capture the phylogenetic scales at which each trait creates the most disparities between species of the community, and so the scales at which species filters primarily occur when communities are assembled (Supplementary Fig. 3). We also show that this phylogenetic scale disparity depends on the model of trait evolution that was used: BDTT correlation profiles pic in deeper regions of the tree when the environmental gradient is defined by a trait that mostly evolved in deep regions of the phylogeny (see red profiles in Supplementary Fig. 3). In conclusion, our simulations show that BDTT is able to discriminate the effect of factors (here environmental gradients) on community dissimilarities along the phylogenetic scale, if these factors are actually related to traits that evolved at different phylogenetic time scales.

Supplementary Note 3. Robustness of the signal of bacterial time scale disparity between the effects of host phylogeny and host diet on microbiome composition

Here, we detail the results obtained in multiple control conditions concerning the robustness of the time scale disparity signal illustrated in Figure 1 of the main text, showing that host phylogeny and diet shape the composition of mammalian gut microbiota at different bacterial phylogenetic scales.

Workflow diagram and control experimental conditions

The main text and Figure 1 present results obtained by using rough time calibrations to slice the phylogenetic community tree and to continuously cluster sequences from the leaves to the root of the tree (see Figure 1, Results – Phylogenetic decomposition of community dissimilarities and Supplementary Fig. 2). We also used branch lengths expressed in substitution/site as a proxy of time in the BDTT approach, to continuously slice the phylogenetic tree and cluster sequences into OTUs, allowing us again to compute β-diversities and to measure correlations with host phylogenetic and dietary distances. Furthermore, we controlled for the influence of the unequal sampling size across samples by performing rarefaction of the OTU tables. We also run BDTT with an alternative rooting of the phylogenetic tree, or by restricting the analysis to Firmicutes or Bacteroidetes sequences only. All the aforementioned control experiments were run with two different ways of measuring the correlation between diet/host

phylogeny distance matrices (see Methods) and either un-weighted (Sørensen) or weighted (Bray-Curtis) compositional dissimilarity matrices.

Impact of rooting, rarefaction & restricting the analysis to Firmicutes and Bacteroidetes

To produce the results presented in Fig. 1A, we used all 16S sequences, the bacterial phylogenetic tree was rooted on Actinobacteria, branch lengths were expressed in "time" units to continuously produce OTU tables along the tree, and, finally, we used non-rarefied OTU tables to compute the β-diversities at each time slice. This particular set of experimental conditions is representative of our general experimental procedure: as explained below, all other combinations of these experimental conditions produced similar results.

Control experiments suggest that the BDTT analysis uncovers a genuine signal of scale disparity and is not affected by systematic biases. Supplementary Figure 4 shows that our findings are robust to various control conditions. Host phylogeny systematically correlates the most with community compositional dissimilarities at recent time scales of bacterial evolution, contrarily to diet, which correlates the most at more ancient time scales. Importantly, we show that using evolutionary distance rather than absolute time as a criterion to continuously slice the tree to cluster sequences into OTUs does not change the original conclusions. As expected, rarefying the data matrices results in noisier correlation profiles with host phylogeny and diet, although the scale disparity signal remains very strong. Finally, our results show that both Firmicutes and Bacteroidetes, the two main phyla present in mammalian gut microbiota, segregate across hosts in the same way with respect to host phylogeny and diet.

Impact of lowly supported branches in the bacterial phylogenetic tree

We make use of the phylogenetic relationships between 16S sequences to create OTUs, using either evolutionary time or evolutionary distance as a criterion to delineate monophyletic groups of sequences (see Supplementary Fig. 2). We evaluated the impact of uncertainties in topology reconstruction on the clustering of OTUs and, so, on the correlation measured at each cut-off used to define slices. In the phylogenetic community tree, we collapsed nodes having a low support (SH-like support below 0.8). We re-defined slices along this new tree and re-run BDTT to measure correlations with host phylogeny and host diet distances. We did not observe

an impact of uncertainties in tree topology on our main conclusions about time scale disparity between host phylogeny and host diet (Supplementary Fig. 4).

Correlation profiles expected under a null model controlling for the effect of the tree structure

Our progressive clustering along the phylogenetic community tree continuously agglomerate sequences and creates larger and larger OTUs that are more and more widely distributed across samples. Consequently, correlations between community dissimilarities and host phylogeny and diet distances could be influenced by the spurious effect of the hierarchical nature of the phylogenetic tree [10], *e.g.* making easier to detect significant correlations at large phylogenetic scales. To ensure that our BDTT profiles and associated host phylogeny/diet correlation profiles are not the result of the hierarchical dependency between leaves and internal branches of the tree, we compared the observed signal with appropriate null expectations. More specifically, we shuffled the names of the leaves of the bacterial community phylogenetic tree and re-run the BDTT analysis. This null model breaks down the observed phylogenetic relationship between sequences/OTUs, but keeps constant the hierarchical structure of the tree. It also conserves the elements of the OTU table deduced from the repartition of unique sequences (tips of the tree) across hosts, so that the correlations between compositional dissimilarities and host phylogeny or diet at the youngest time slice remain identical to the observed ones. However, we observed that under this null model, correlations drop immediately when considering older time scales (Supplementary Fig. 5). We conclude that the observed correlation profiles capture a biological signal and are not the result of an artefact due to the hierarchical structure of the bacterial tree.

No influence of the intra host-species variability in microbiome compositions on our conclusions drawn from inter host-species analyses

Several studies have shown that the variability in microbiome compositions within a mammalian host species is on average lower than the amount of compositional differences between individuals belonging to different host species [11-15]. However, the choice of the representative individuals for each host species that we have selected may be biased towards outliers by chance, which could bias all of our results. We controlled for the impact of intra-host

species variability in microbiome compositions on our conclusions as follows. The original dataset by Muegge et al. [16] includes a couple of individuals for 7 hosts (baboon, big horn, human, chimp, hyrax, lion and okapi). We have substituted these 7 individuals with their conspecific and we have re-processed the data with the exact same procedure. We computed the BDTT profiles characterizing the correlation between the new microbiome compositional dissimilarities and host phylogenetic or dietary distances (which remain unchanged). Interestingly, the new individuals for hyrax, baboon and big horn were not sampled in the same zoo as the original individuals that we selected. Supplementary Fig. 6_shows that our initial conclusions regarding how host phylogeny and diet shape microbiome compositions over evolutionary time hold true with this different host sampling, further confirming that intra-host compositional variability does not blur signals of inter-host compositional differences.

The difference in granularity between the host phylogenetic and dietary distance matrices does not explain the scale disparity between the effects of the two factors

Compared to the matrix of host phylogenetic distances, the matrix of host dietary distances is much more coarse-grained (see Methods – Host phylogeny and dietary data). For this reason, one might expect that this difference in granularity impact the shape of the correlation profiles and create a spurious signal of scale disparity with correlations between microbiome composition and host phylogeny being higher in recent regions of the bacterial tree and correlations with host diet being higher with more ancient bacterial groups. If this is true, using coarse-grained host phylogenetic distance matrices should displace the area where the correlation with host phylogeny is maximum towards more ancient regions of the bacterial tree, just as we observe for diet. We have controlled for this as follows. We re-ran BDTT using coarse-grained distance matrices for host phylogeny. We used a set of host phylogenetic distance thresholds to define several host phylogenetic distances matrices, with all pairwise distances below these thresholds set to null distances. Supplementary Fig. 7 shows that the correlation with host phylogeny globally decreases when using less and less informative predictors. However, even with coarse-grained host phylogenetic distance matrices, the correlation with host phylogeny is always localized at recent time scales on the bacterial phylogeny, separated from the highest correlations with host diet along the phylogeny of bacteria. This control experiment further confirms that host phylogeny and diet impact gut microbiome compositions at different bacterial

phylogenetic scales, and that these effects can be partitioned with our BDTT approach, which is illustrated in Figure 1A.

The contribution of diet on microbiome compositions is partially decoupled from the host phylogenetic history

We asked whether simulated traits evolving along the host phylogeny in the same way as diet does would produce BDTT correlation profiles that are similar to the correlation profile obtained when correlating observed diets with gut microbiome compositions. This control experiment will give us a sense of how likely we are to selectively detect correlations at particular timescales as a result of a particular tempo of trait evolution that records the phylogenetic history of hosts (although diet and host phylogeny are poorly correlated at the scale of our 33 mammals (Mantel test, $R^2 = 0.01$, *p-value* = 0.09), diet is locally correlated with host phylogeny). If we detect a peak in correlation between these simulated traits and microbiomes at a timescale similar to the one detected with observed diets, it would be evidence that the correlations with diet would only represent an artefact of the phylogenetic inertia that diet carries and not a 'true' effect of diet itself.

We simulated phylogenetically-conserved traits that evolved along the mammalian phylogeny at the same rate as diet does. We have estimated the transition rates between dietary states (herbivory, carnivory and omnivory) with the ARD (All Rates Different) markovian model (implemented in the ape R package) along the phylogeny of 1,534 mammals that we have used elsewhere in the paper (note that the ARD model was selected because it is the model that best fits the data among all models that we have tested). We used the ML estimates of these transition rates to simulate traits along our phylogeny of 33 mammals, so that each trait is forced to evolve at the same rate as diet does along the host phylogeny. We ran 100 simulations. We used these simulated traits to build distance matrices that have the same coarse granularity as the dietary distance matrix computed from the observed diet. We used these dietary distance matrices to run BDTT, and we compared the simulated correlation profiles with the correlation profile obtained when using observed diets (note that, for the sake of fair comparisons, correlations with observed diets were computed with the same trait categories (herbivory, carnivory and omnivory), and not the nine categories (see Supplementary Table 1) used in Fig. 1A and Supplementary Fig. 4). Supplementary Fig. 8 shows that the simulated traits poorly predict the compositional

dissimilarities of our mammalian gut microbiomes. More importantly, we do not observe any increase in explanatory power when computing correlations at ancient time scales, ruling out the possibility that the peak of correlation with observed diet at ancient time scales is simply an echo of phylogenetic history written in diet (or only driven by the coarse granularity of the dietary distance matrix). It further supports the claim that there is an effect of diet that is independent from the host phylogeny (see Results – Phylogeny and diet shape microbiomes at different scales).

Supplementary Note 4. The distribution of individual bacterial lineages confirms the scale disparity between the effects of host phylogeny and diet.

We used PERMANOVA to correlate the presence/absence pattern of all bacterial lineages in the phylogenetic tree of all 16S sequences (*i.e.* all monophyletic clades defined by all internal nodes) to host phylogenetic and dietary distances. We show that the individual lineage-level signal as captured by PERMANOVA tests recapitulates the aggregate signal measured at the community scale with Mantel or GDM tests (compare pie-charts of Figure 1C in the main text with correlation profiles of Fig. 1A). Notably, we recover the fact that host phylogeny and host diet drive the composition of gut microbiota at disparate phylogenetic scale of bacterial evolution.

Supplementary Note 5. Host phylogeny and diet mostly influence the distribution of non-overlapping bacterial lineages

Bacterial lineages correlating with host phylogeny and diet lowly overlap

We used a second approach to measure to what extent host phylogeny and diet affect the distribution of overlapping bacterial lineages across hosts. The difference of phylogenetic scale at which host phylogeny and diet shape the composition of gut microbiota may emerge from two different patterns: as diet discriminates at high phylogenetic scales, recent bacterial lineages correlated to host phylogeny may or may not be be nested in the more ancient diet-related bacterial lineages. In the latter case, the bacterial lineages targeted by host phylogeny and diet are non-overlapping. Supplementary Fig. 9a illustrates the two possible scenarios. To distinguish between these two hypotheses, we proceeded as follows: at the slice S_{max} where diet correlates the most with compositional dissimilarities, we detected the individual lineages correlated to diet

according to PERMANOVA tests (after controlling for multiple comparisons using FDR), and then removed these diet-related ancient lineages from the tree. Then, we re-ran the BDTT analysis and measured correlations with host phylogeny. We found that removing these ancient lineages erased the signal of correlation between community compositional dissimilarities and diet at all phylogenetic scales (Supplementary Fig. 9b), demonstrating that lineage-level and community-level (BDTT) analyses show congruent results. Interestingly, we found that removing these ancient lineages did not erase the signal of correlation between gut microbiota composition and host phylogeny (Supplementary Fig. 9b). This suggests that most of the host phylogeny-related bacterial lineages are non-overlapping with (and not nested within) the host diet-related bacterial lineages. Nevertheless, we observe that the correlation signal with host phylogeny is slightly weaker. We tested whether this slight difference was significant in comparison with a null expectation. With the original phylogenetic tree, a number *K* of lineages are nested within the ancient lineages that correlate with diet at the slice S_{max}. The null model randomly selects and discards *K* OTUs from the OTU table at each phylogenetic slice, and then the correlation with host phylogeny is measured. 100 iterations are performed at each slice, providing a null distribution of R^2 values. We observe that the decrease in correlation with host phylogeny is slightly higher than randomly expected under a null model. This result indicates that, while most of the clades correlating with host phylogeny and host diet are non-overlapping, there is a significant portion of derived lineages correlating with host phylogeny at recent scales that are nested within more ancient clades correlating to host diet. We estimate that, on average, 8% of lineages related to host phylogeny per slice are nested in higher clades correlated to diet.

<u>The covariance between host phylogenetic and dietary distances poorly explains community dissimilarities</u>

The intersection variance informs if the covariance between the two predictors (host phylogeny and diet) explains community composition. We observe that at recent time scales, there is a weak intersection correlation (between 0.03 and 0.04), which then progressively drops when larger OTUs are progressively defined at higher phylogenetic scales (Supplementary Fig. 9c). It suggests that the effects of host phylogeny and diet on gut microbiota are largely decoupled, and confirms that these two factors mostly define non-overlapping niches (see

‘Bacterial lineages correlating with host phylogeny and diet lowly overlap’ in Supplementary Note 5).

Supplementary Note 6. BDTT reveals functional differentiations in bacterial lineages

The OMI analysis allows us to jointly represent hosts separated by diet distances and their bacterial lineages that correlate to host diet on the same ordination space. This technique computes the niche breadth of each lineage with respect to the environmental gradient, here represented by host diet. We observed that bacterial lineages exhibit niche breadth that encompass carnivorous or herbivorous mammals only, highlighting their functional specificities with respect to each diet category (Figure 2A). Focusing on the phylogenetic relationships of each of these lineages with respect to their most-closely related lineages, we observed clear patterns of functional differentiations between the types of guts that can be colonized, in reference to host diet. Some bacterial lineages exhibit a niche breadth encompassing carnivorous hosts only, such as particular lineages belonging to *Lactobacillus* or *Coriobacterineae*, while others have niches restricted to herbivorous guts, such as specific *Verrucomicrobiaceae* or *Bacteroidales* lineages. However we did not observe ‘omnivore-specific’ bacterial lineages (see Results – Omnivores do not harbour omnivore-specific bacteria). In Fig. 2A, one bacterial lineage, an Enterobacteriaceae, is present in a central position, close to omnivores (violet square). However, this lineage has a large niche breadth and is not omnivorous-specific. While it is observed in several omnivores, it is also observed in carnivores, such as lions and polar bears and in an herbivore, such as giraffes. Taken together, these results suggest that herbivorous and carnivorous guts contain specialist diet-related bacterial lineages, contrary to omnivores that filter bacterial lineages from these more circumscribed herbivorous and carnivorous collections.

Supplementary Note 7. Reconstructing ancestral diets in mammals

<u>Accuracy of our microbiome-based method of diet prediction</u>

Our microbiome-based inference of diet uses a PCA to cluster hosts according to their microbiome compositional differences into a subspace defined by linearly uncorrelated variables called principal components, or axes. The variables are defined so that the first axis represents the highest possible variance in the data, and subsequent axes are defined as having the highest

possible variance while being orthogonal to the preceding axis. We use these axes as explanatory variables in a multiple logistic regression model, which allows us to predict diet. In order to determine the appropriate number of PCA components (axes) to integrate in the logistic regression, and to avoid overfitting issues, we used the AIC criterion to penalize the optimized likelihood of the regression model. We show that using the first 3 axes represent the best situation between the goodness of fit and the complexity of the regression model (Supplementary Fig. 10a-c). In all subsequent analyses, we used the 3 first axes of the PCA to optimize the multinomial logistic regression models.

We evaluated the predictive power (accuracy) of our microbiome-based method of diet inference. We performed cross-validation experiments to evaluate to what extent the method is able to accurately predict diets of extant mammals. We defined two types of datasets: the training dataset, which randomly contains 80% or 90% of the 33 mammalian microbiome, and the *testing* dataset, which contains the remaining 20% or 10% of microbiome, respectively. We performed 100 random replications. The goal of this cross-validation experiment is to predict the diet of the testing dataset using our predictive model trained with the training dataset, providing a measure of the ability of the model to generalize to independent datasets. We used a probability cut-off of 0.5 to assign a diet to a prediction. We show that on average, the model is able to accurately predict diet categories in 78% and 80% of cases with the 80% and 90% training datasets, respectively (Supplementary Fig. 10b-c).

<u>Microbiome-based and trait-based inference of ancestral diets in mammals</u>

Inferences of ancestral diets along the mammalian phylogeny are represented in Supplementary Fig. 10d-f. Three different reconstructions were performed: one with our microbiome-based method and two trait-based analyses with a model of trait evolution. The two trait-based reconstructions were performed either with a poor taxonomic sampling (the 33 mammalian species for which we also have microbiome compositions) or with a rich taxonomic sampling (1,534 mammals [17]) that is representative of the extant mammalian diversity. We used the trait reconstruction with a rich taxonomic sampling as our reference against which we measure the predictive power of the trait-based and microbiome-based reconstruction method on the poor taxonomic sampling. First, we measure the accuracy of both methods (i.e. the ability of

the two methods to reconstruct diets similar to our reference). Second, we measure the precision of each reconstruction.

In comparison with the best trait-based reconstruction performed with 1,534 mammals [17] that we consider as a reference, both the microbiome-based method and the trait-based method using 33 mammals predict 70% of similar ancestral dietary categories. With the microbiome-based approach, divergences appear for the most ancestral mammalian ancestors, for which paleontological evidence and previously published trait-based inferences using 1,534 mammals suggest carnivorous ancestors [17,18]. The main reason why the microbiome-based method is not able to recover a signal for ancestral carnivory likely lies in the poor taxonomic sampling of carnivorous mammalian clades that branch deeply in the mammalian tree (carnivorous Chiroptera, Afrotheria, Xenarthra and Marsupialia) and the low bacterial richness of Carnivora gut microbiome. Indeed, as carnivorous mammals are restricted to the Carnivora clade and in the Armadillo branch in our 33 mammals dataset, and as carnivores contain very specific bacterial lineages in their gut that are not found in other non-carnivorous guts, the most likely reconstruction of ancestral microbiome involves gains of these carnivorous-specific lineages in the ancestor of Carnivora and in the Armadillo lineage (see Supplementary Fig. 10d-f), and not in most ancestral mammalian lineages.

We then focused on the measurement of the precision of ancestral reconstructions. Interestingly, no ancestor is associated to a uniform, non-informative distribution of probabilities for each diet category (*e.g.* [0.33; 0.33; 0.33] for herbivory, omnivory and carnivory, respectively) when diets are reconstructed with the microbiome-based method. Probability distributions are often concentrated around a single diet category, highlighting the capacity of the microbiome-based method to provide precise inferences (see Results – Gut microbiomes can predict ancient mammalian diets).

Here, the mean entropy for the trait-based reconstruction preformed with 1,534 mammals is 0.23, showing that predictions are precise. The mean entropy with 33 mammals and the microbiome-based reconstruction is 0.43. This higher entropy means that there is more uncertainty in the prediction of diets. However, the mean entropy with a similar taxonomic sampling (33 mammals) and the trait-based reconstruction is 0.56, significantly higher than the

microbiome-based average entropy (*p-value* < *0.001*). It shows that with a similar taxonomic sampling, the microbiome-based method is more precise. Note that this higher precision for the microbiome-based method is not associated with inaccurate predictions, when considering trait-based reconstructions with the 1,534 mammals as reference. For the 30% of nodes at which the microbiome-based method predicts a different diet, the average entropy is 0.51, higher than when all nodes are considered (0.43), meaning that the coherent diet predictions tend to be associated with higher precisions than the more unlikely predictions.

Altogether, all our results suggest that gut microbiome compositions bring valuable phylogenetic information for ancestral diet reconstruction in addition to the single discretized diet character (H, O or C).

<u>Reconstruction of ancient diets from microbiomes defined at alternative bacterial phylogenetic scales</u>

We selected the 300 Ma time slice to phylogenetically cluster 16S sequences into OTUs and to determine microbiome compositions to infer ancestral diets for two reasons. First, this time slice is ancient enough to define some clusters of sequences that correlate with diet (at more recent time slices (shallow phylogenetic scales), no or very few OTUs correlate to diet (see Fig 1A, 1D). However, the 300 Ma slice is recent enough to possess bacterial lineages (OTUs) that exhibit distributions across hosts that are restricted to either carnivorous or herbivorous mammals (at too high taxonomic levels, all bacterial lineages that correlate to diet are herbivorous-specific, see Fig. 1D). The 300 Ma slice defines OTUs that have on average ~94% of 16S DNA similarity. We tested whether the reconstruction of diets is impacted by the taxonomic resolution at which gut microbiome compositions are determined, using a more recent (with OTUs having ~97% of 16S similarity) and more ancient time slice (with OTUs having ~91% of 16S similarity).

We observed that ancestral diet reconstructions are less accurate when defining communities at these shallower or higher phylogenetic scales. With OTUs having ~97% similarity, the accuracy drops at 60% (vs. 70% with the 300 Ma time slice, *z*-score test, *p-value* = 0.4). This decrease is expected since no OTU significantly correlates with diet after correcting for multiple tests (see Methods). Even though we performed this reconstruction with this recent time slice, it is theoretically not wise and accurate, because a lot of OTUs correlate with host phylogeny, making the reconstruction of ancient diets possibly biased by the confounding effect

of host phylogeny. At the more ancient 600 Ma time slice (OTUs with ~91% similarity), the accuracy of diet reconstruction drops from 70% to 41% (*z*-score test, *p-value* = 0.02*). The reason lies in the absence of carnivorous-specific bacteria when microbiome compositions are determined at this higher phylogenetic time scale. Most of bacterial lineages that were present in carnivorous mammals at more recent phylogenetic scales are now also observed in omnivorous mammals. Consequently, the correlation between diet and extant microbiome compositions is weaker, making the recovery of the signal for ancient adaptations to carnivorous diets from reconstructed microbiome compositions impossible.

Together, these alternative reconstructions demonstrate that information on extant and past diets is limited in a confined range of bacterial taxonomic/phylogenetic scales. Thus, by progressively constructing communities at fine-grained resolutions all along the bacterial phylogenetic tree, our BDTT approach allowed us to detect and analyse these signals to reveal the complex patterns of host-microbiome evolution related to diet.

Supplementary Note 8. Convergent acquisitions of bacterial lineages and adaptation to herbivory

We observe that multiple convergent acquisitions of herbivorous-specific lineages (having an ecological niche that only encompasses herbivorous guts, see the OMI analysis in Fig. 2) are associated to dietary transitions towards herbivory, either from an omnivorous or carnivorous diet (see Supplementary Fig. 11). Most of the convergent acquisitions concern transitions towards herbivory, and partly explain why herbivores collectively have the richest gut bacterial communities [11]. Using our probabilistic model of gain and loss of lineages (see section 1.7) to reconstruct ancestral communities, we observed that these herbivorous-specific bacterial lineages have a higher rate of horizontal acquisition than the rest of the lineages (*p-value* < 0.01), highlighting the ability of these bacterial lineages to colonize many different herbivorous hosts. Note that this signal could not have been recovered without our scale disparity analysis that we performed with BDTT, as these bacterial lineages segregate more randomly across hosts at shallower phylogenetic levels. Our results also show bacterial convergent adaptations in the ability to degrade herbivorous-derived compounds like complex polysaccharides across different bacterial phyla, such as Verrucomicrobia, Firmicutes, Bacteroidetes and Actnibacteria (see OMI in Fig. 2 and Supplementary Fig. 11). Finally, we observe that in several cases, we predict

parallel transitions towards herbivory in sister mammalian lineages: African Elephant and Hyrax, Ring-Tailed Lemur and Black Lemur and Colobus and Baboon (Supplementary Fig. 10d-f). It suggest that each mammalian lineage in these pairs possess some specific bacterial lineages that are herbivorous-specific, and which are not present in the sister mammalian lineage. In two out of these three cases (african elephant and hyrax and colobus and baboon), the trait-based reconstruction with 1,534 mammals predicts the same convergent adaptations to herbivory. This reconstruction uses higher phylogenetic information since it uses additional lineages branching around these three couples of lineages. However, the trait-based reconstruction with 33 mammals fails at recovering these parallel adaptations for the african elephant and hyrax, and the colobus and Baboon.

Supplementary Note 9. Variation of co-speciation rates across the bacterial phylogenetic tree and associations with IBD

We have controlled whether the mammalian gut sequences used to test for patterns of co-speciation in our study are also readily observed in environmental samples, using BLAST analyses. We obtained data from two previous studies in which the V2 16S region was also sequenced from soil and aquatic samples. The soil data encompasses both forest and grassland soils [19]. The aquatic samples are from surface oceanic waters and were sampled over three years [20]. We trimmed sequences that had low quality scores using Mothur [21], and only retained unique sequences. For the two environmental datasets, the average read length is similar to the length of our mammalian sequences (~250bp). We observed that the amount of mammalian unique sequences that are identical to these environmental sequences is very low: 0.1% (6 out of 44,444 mammalian reads) for the aquatic samples, and 0.15% for the soil samples. We have also used a less conservative approach, allowing blast similarities to range between 99% and 100%, with a minimum alignment coverage of 90%. Only 0.3% of mammalian sequences have similarities with environmental sequences according to these criteria, and only 2% when compared to soil sequences.

This control confirms that our large-scale signal of co-speciation, which implies multiple bacterial lineages, is not affected. It also confirms previous reports showing that mammals are hosts of very specific gut bacterial communities, which lowly overlap in composition with environmental communities [22,23].

Interestingly, bacterial lineages harboring high co-speciation rates are distributed across all major bacterial phyla that are present in mammalian guts (Firmicutes, Bacteroidetes and Actinobacteria) (see Supplementary Fig. 13a). However, they are not randomly distributed within these phyla. Some taxonomic groups are enriched in OTUs that co-speciate more than they swap from host to host (Supplementary Fig. 13a). For example, at the rank of taxonomic orders, Selenomonadales and Cytophagales-like OTUs have an average co-speciation rate 0.81 and 0.76, respectively (Supplementary Fig. 13b). Note that Cytophagales were previously reported as a bacterial order being present in environmental samples [24]. Here, all our 16S Cytophagales-like sequences have low RDP assignation probabilities to this rank (~ 0.2), meaning that they represent gut-adapted distantly related lineages to those previously characterized and that are present in 16S databases. We can safely exclude potential contaminations from the environment, since our Cytophagales-like OTUs are all present in multiple (≥ 4) hosts. Contrary to these orders that have high occurrence of co-speciating OTUs, others like Lactobacillales or Desulfurovibrionales have OTUs with low co-speciation rates (0.17 and 0.11 on average, respectively) (Supplementary Fig. 13b). These large heterogeneities of co-speciation rates across bacterial lineages were also observed at finer taxonomic resolutions: at the family (Supplementary Fig. 13c) and at the genus (Supplementary Fig. 13d) levels. However, note that using these taxonomic phylotypes might overshadow intra-specific heterogeneities of the amount of vertical inheritance, *i.e.* when a single bacterial lineage that is highly vertically inherited branches next to lineages that can more freely being transmitted from host to host or being acquired from the environment. These scenarios might represent potential adaptations making them more intimately associated with the host, and are better illustrated in Supplementary Fig. 13a, where no *a priori* taxonomic clustering is performed.

Overall, very little is known about the bacterial genera that have the highest average co-speciation rates (Figure 4F and Supplementary Fig. 13d). Most of the 16S sequences belonging to these OTUs are assigned to taxonomic ranks with low confidence, according to the RDP classifier. It means that these bacterial lineages are mostly under-investigated, and have no close phylogenetic relationship with known sequences present in databases. Here, we propose to briefly present and discuss the current and limited knowledge on some of these genera. For instance,

Rikenella-like organisms were observed in the gut of various organisms, from leech to human [25]. However, very few strains were isolated in pure culture, limiting our knowledge on their characteristics and on the nature of their interactions with hosts. However, it was noted that some *Rikenella*-like lineages display tight historical associations with their leech hosts and that some degrees of topological congruence between *Rikenella*-like and host phylogenies were observed, suggestive of vertical inheritance [25]. Some bacterial lineages belonging to the *Sporobacter* genus were observed in the intestinal tract of social termites [26,27], which were strongly suggested to vertically transmit their gut microbiome from one generation to another [28-30]. Finally, some studies focused on the characterization of *Mitsuokella*, a bacterial genus belonging to Bacteroidetes. *Mitsuokella* are phytase-producing bacteria that were previously observed in the gut of several host species, from pigs, to cattle to humans [31-34]. Overall, our results highlight the diversity of interesting bacterial lineages that remain to be characterized and studied to further understand the complex interactions between hosts and their gut microbiome. It opens up exciting new avenues of research, which may help to reveal the functions of these organisms and how, and to what extent, natural selection operates at the level of their interaction with hosts.

In a recent study [35], the *gyrB* gene was amplified in three different families (Bacteroidaceae, Bifidobacteriaceae and Lachnospiraceae) and was used to search for patterns of co-speciation in four hominid hosts, humans, chimpanzees, bonobos and gorillas. These authors have shown that Bacteroidaceae and Bifidobacteriaceae possess some lineages that harbor patterns of co-speciation. However, these authors have also found that Lachnospiraceae lineages have lower co-speciation rates and have experienced several host-swaps along Hominid evolution. On average, our results show that both Bacteroidaceae and Lachnospiraceae OTUs have low co-speciating rates (Supplementary Fig. 13c; there was not enough sequenced Bifidobacteriaceae to investigate patterns of co-speciation/host-swap). It is difficult at this stage to make cross-study comparisons, as we do not know how the primers used to amplify the 16S gene compare to those used to amplify *gyrB* with respect to their ability to capture bacterial diversity. In addition, the host evolutionary time scales at which our investigations were carried out are vastly different. Future investigations are needed to further characterize the rates of co-speciation across different bacterial taxa and across all mammalian hosts.

In total, 20 OTUs belonging to 10 different genera are observed to be highly co-speciating with hosts and, at the same time, being present in humans (*Anaerotruncus*, *Subdoligranulum*, *Sporobacter*, *Oscillibacter*, *Butyricicoccus, Lachnospiracea incertae sedis*, *Blautia*, *Clostridium XlVa*, *Coprococcus*, and *Anaerofilum*). Remarkably, we found that 13 out of these 20 OTUs belong to 5 bacterial genera that were previously shown [36] to strongly correlate with Crohn's Disease (CD) and Ulcerative Colitis (UC), two pathologies termed inflammatory bowel diseases (IBD). IBD provokes inflammation in the gut through the ulceration of the intestinal mucosa, and is currently incurable. These genera are *Anaerotruncus*, *Subdoligranulum*, *Sporobacter*, *Oscillibacter*, and *Butyricicoccus*. In our previous study [36], we compared the gut microbiome of non-IBD vs. IBD patients and we showed that these five genera are largely depleted in patients affected both by CD and UC (Supplementary Fig. 14). We tested whether gut bacteria with high co-speciation rates are more frequently part of genera associated with IBD in comparison with lowly-co-speciating OTUs. We performed a permutation test, shuffling co-speciation rates across OTUs, and counting for each replicate the number of highly co-speciating OTUs (rate > 0.8) that falls within genera associated with IBD. We then compared the observed number of highly co-speciating OTUs associated with IBD (13) to this random distribution. We observed that this enrichment is strongly significant (p-value = 0.00149).

These correlations between co-speciation and gut-related pathologies are intriguing. We can speculate that bacterial lineages that are tightly associated with hosts to the point of being vertically inherited over millions of years of host evolution are functionally important for the host and may influence its health and physiology. Future studies are needed to turn these suggestive and exciting correlations to proper demonstration of causation.

Supplementary Discussion

Other datasets containing amplicons of 16S rDNA genes extracted from gut microbiome of specific mammalian clades are available in the literature and were not included in our study (see [13,37] for instance). However, several reasons prompted us not to include these data to the dataset of Muegge and colleagues [16]. First, we wanted to avoid biases introduced with variation in DNA extraction protocols, choice of 16S primers and sequencing platform. More importantly, 16S reads in the Muegge dataset [16] are amplicons of the V2 region of the 16S rDNA gene. The other datasets that were published later targeted other 16S regions, especially the V4 region [37]. As our approach relies on an alignment of 16S reads to construct the bacterial phylogenetic tree in order to cluster sequences into OTUs and to measure β-diversities, only homologous regions of the 16S sequence could be considered. This excludes *de facto* the use of different 16S regions. Furthermore, the number of hosts in the dataset of Muegge et al. [16] (33 mammals) do not preclude us from obtaining strong statistical power: all our quantitative evaluations show that measured explained variances (R2) are high, and that statistical tests with respect to null models in Figure 1, Figure 3 and Figure 4 are highly significant.

Our phylogenetic analyses support co-speciation as playing a significant role in the diversification of mammalian gut symbionts relative to a null model of random associations. However, the extent to which these associations can be further investigated are limited by bounds imposed by both the 16S marker gene. Furthermore, other factors that are locally correlated with host phylogeny might contribute to generate some of the patterns of topological congruency between symbiont and host phylogenetic trees. For instance, fine-grained differences in diet that are not captured by our measurement of dietary distances could drive adaptations and functional differentiation of some of the bacterial lineages that we analysed, which could result in patterns of diversification mimicking host diversification. However, such cases would only appear if dietary distances match host phylogenetic distances. It has been shown that classic models of trait evolution (Brownian Motion or Ornstein–Uhlenbeck processes) cannot reproduce such patterns of intimate coevolution between host phylogeny and trait (*e.g.* diet) [38,39]. Only very constrained models of trait evolution may generate phylogenetic and trait information that are congruent, *e.g.* when competitive interactions strongly occurs within communities of animal species [39]. Alternatively, the iterative bacterial specialization on related host linages (or host-swap

speciation) can yield concordant symbionts/host topologies even in the absence of co-speciation [40]. Consequently, although our study provides compelling evidence that congruence with host topology is intriguingly correlated with evolutionarily and functional aspects of the gut microbiome, additional studies using other bacterial phylogenetic markers and larger taxonomic samplings of hosts will provide more details on the mechanistic origin and precise timing of this topological concordance.

Supplementary References

1. Britton, T., Anderson, C. L., Jacquet, D., Lundqvist, S. & Bremer, K. Estimating divergence times in large phylogenetic trees. *Systematic Biology* **56,** 741–752 (2007).
2. Schirrmeister, B. E., de Vos, J. M., Antonelli, A. & Bagheri, H. C. Evolution of multicellularity coincided with increased diversification of cyanobacteria and the Great Oxidation Event. *Proc Natl Acad Sci U S A* **110,** 1791–1796 (2013).
3. Parfrey, L. W., Lahr, D. J. G., Knoll, A. H. & Katz, L. A. Estimating the timing of early eukaryotic diversification with multigene molecular clocks. *Proc Natl Acad Sci U S A* **108,** 13624–13629 (2011).
4. Brocks, J. J. *et al.* Biomarker evidence for green and purple sulphur bacteria in a stratified Palaeoproterozoic sea. *Nature* **437,** 866–870 (2005).
5. Blomberg, S. P., Garland, T. & Ives, A. R. Testing For Phylogenetic Signal in Comparative Data: Behavioral Traits Are More Labile. *Evolution* **57,** 717–745 (2003).
6. Harmon, L. J. *et al.* Early bursts of body size and shape evolution are rare in comparative data. *Evolution* **64,** 2385–2396 (2010).
7. Münkemüller, T. & Gallien, L. VirtualCom: a simulation model for eco- evolutionary community assembly and invasion. *Methods in Ecology and Evolution* **6,** 735–743 (2015).
8. Lozupone, C. & Knight, R. UniFrac: a new phylogenetic method for comparing microbial communities. *Applied and Environmental Microbiology* **71,** 8228–8235 (2005).
9. Cavender-Bares, J. & Reich, P. B. Shocks to the system: community assembly of the oak savanna in a 40- year fire frequency experiment. *Ecology* **93,** S52–S69 (2012).
10. Hardy, O. J. Testing the spatial phylogenetic structure of local communities: statistical performances of different null models and test statistics on a locally neutral community. *Journal of Ecology* **96,** 914–926 (2008).
11. Ley, R. E. *et al.* Evolution of mammals and their gut microbes. *Science* **320,** 1647–1651 (2008).
12. Ochman, H. *et al.* Evolutionary Relationships of Wild Hominids Recapitulated by Gut Microbial Communities. *Plos Biol* **8,** e1000546–8 (2010).
13. Moeller, A. H. *et al.* Rapid changes in the gut microbiome during human evolution. *Proc Natl Acad Sci U S A* **111,** 16431–16435 (2014).
14. Sanders, J. G. *et al.* Baleen whales host a unique gut microbiome with similarities to both carnivores and herbivores. *Nature Communications* **6,** 8285 (2015).
15. Bik, E. M. *et al.* Marine mammals harbor unique microbiotas shaped by and yet distinct from the sea. *Nature Communications* **7,** 10516 (2016).
16. Muegge, B. D. *et al.* Diet Drives Convergence in Gut Microbiome Functions Across Mammalian Phylogeny and Within Humans. *Science* **332,** 970–974 (2011).
17. Price, S. A., Hopkins, S. S. B., Smith, K. K. & Roth, V. L. Tempo of trophic evolution and its impact on mammalian diversification. *Proc Natl Acad Sci U S A* **109,** 7008–7012 (2012).
18. Stevens, C. E. & Hume, I. D. *Comparative Physiology of the Vertebrate Digestive System*. (Cambridge University Press, 2004).
19. Nacke, H. *et al.* Pyrosequencing-based assessment of bacterial community structure along different management types in German forest and grassland soils. *PLoS ONE* **6,** e17000 (2011).
20. Campbell, B. J., Yu, L., Heidelberg, J. F. & Kirchman, D. L. Activity of abundant and rare

bacteria in a coastal ocean. *Proc Natl Acad Sci U S A* **108,** 12776–12781 (2011).
21. Schloss, P. D. *et al.* Introducing mothur: open-source, platform-independent, community-supported software for describing and comparing microbial communities. *Applied and Environmental Microbiology* **75,** 7537–7541 (2009).
22. Ley, R. E., Peterson, D. A. & Gordon, J. I. Ecological and Evolutionary Forces Shaping Microbial Diversity in the Human Intestine. *Cell* **124,** 837–848 (2006).
23. Hacquard, S. *et al.* Microbiota and Host Nutrition across Plant and Animal Kingdoms. *Cell Host and Microbe* **17,** 603–616 (2015).
24. Reichenbach, H. in *The Prokaryotes* 549–590 (Springer New York, 2006). doi:10.1007/0-387-30747-8_20
25. Siddall, M. E., Min, G.-S., Fontanella, F. M., Phillips, A. J. & Watson, S. C. Bacterial symbiont and salivary peptide evolution in the context of leech phylogeny. *Parasitology* **138,** 1815–1827 (2011).
26. Grech-Mora, I. *et al.* Isolation and Characterization of Sporobacter termitidis gen. nov., sp. nov., from the Digestive Tract of the Wood-Feeding Termite Nasutitermes lujae. *International Journal of Systematic and Evolutionary Microbiology* **46,** 512–518 (1996).
27. Schmitt-Wagner, D., Friedrich, M. W., Wagner, B. & Brune, A. Phylogenetic diversity, abundance, and axial distribution of bacteria in the intestinal tract of two soil-feeding termites (Cubitermes spp.). *Applied and Environmental Microbiology* **69,** 6007–6017 (2003).
28. Dietrich, C., Köhler, T. & Brune, A. The cockroach origin of the termite gut microbiota: patterns in bacterial community structure reflect major evolutionary events. *Applied and Environmental Microbiology* **80,** 2261–2269 (2014).
29. Abdul Rahman, N. *et al.* A molecular survey of Australian and North American termite genera indicates that vertical inheritance is the primary force shaping termite gut microbiomes. *Microbiome* **3,** 5 (2015).
30. Brune, A. & Dietrich, C. The Gut Microbiota of Termites: Digesting the Diversity in the Light of Ecology and Evolution. *Annu. Rev. Microbiol.* **69,** 145–166 (2015).
31. Mitsuoka, T., Terada, A., Watanabe, K. & Uchida, K. Bacteroides multiacidus, a New Species from the Feces of Humans and Pigs. *International Journal of Systematic and Evolutionary Microbiology* **24,** 35–41 (1974).
32. Shah, H. N. & Collins, M. D. Reclassification of Bacteroides multiacidus (mitsuoka, terada, watanabe and uchida) in a new genus Mitsuokella, as Mitsuokella multiacidus comb. nov. *Zentralblatt für Bakteriologie Mikrobiologie und Hygiene: I. Abt. Originale C: Allgemeine, angewandte und ökologische Mikrobiologie* **3,** 491–494 (1982).
33. Lan, G. Q., Ho, Y. W. & Abdullah, N. Mitsuokella jalaludinii sp. nov., from the rumens of cattle in Malaysia. *Int J Syst Bacteriol* **52,** 713–718 (2002).
34. Wang, F. *et al.* Gut Microbiota Community and Its Assembly Associated with Age and Diet in Chinese Centenarians. *J. Microbiol. Biotechnol.* **25,** 1195–1204 (2015).
35. Moeller, A. H. *et al.* Cospeciation of gut microbiota with hominids. *Science* **353,** 380–382 (2016).
36. Papa, E. *et al.* Non-Invasive Mapping of the Gastrointestinal Microbiota Identifies Children with Inflammatory Bowel Disease. *PLoS ONE* **7,** e39242–12 (2012).
37. Delsuc, F. *et al.* Convergence of gut microbiomes in myrmecophagous mammals. *Mol Ecol* **23,** 1301–1317 (2013).
38. Letten, A. D. & Cornwell, W. K. Trees, branches and (square) roots: why evolutionary relatedness is not linearly related to functional distance. *Methods in Ecology and Evolution*

6, 439–444 (2015).
39. Nuismer, S. L. & Harmon, L. J. Predicting rates of interspecific interaction from phylogenetic trees. *Ecol Lett* **18,** 17–27 (2015).
40. de Vienne, D. M. *et al.* Cospeciation vs host-shift speciation: methods for testing, evidence from natural associations and relation to coevolution. *New Phytol* **198,** 347–385 (2013).
